# Protocol for SUM-PAINT spatial proteomic imaging generating neuronal architecture maps in rat hippocampal neurons

**DOI:** 10.1016/j.xpro.2025.103637

**Published:** 2025-03-05

**Authors:** Eduard M. Unterauer, Eva-Maria Schentarra, Kristina Jevdokimenko, Sayedali Shetab Boushehri, Carsten Marr, Felipe Opazo, Eugenio F. Fornasiero, Ralf Jungmann

**Affiliations:** 1 Faculty of Physics and Center for Nanoscience, Ludwig Maximilian University, Munich, Germany; 2 Max Planck Institute of Biochemistry, Martinsried, Germany; 3 Institute of Neuro- and Sensory Physiology, University Medical Center Göttingen, Göttingen, Germany; 4 Institute of AI for Health, Helmholtz Zentrum Munich – German Research Center for Environmental Health, Neuherberg, Germany; 5 Data & Analytics, Pharmaceutical Research and Early Development, Roche Innovation Center Munich, Penzberg, Germany; 6 Department of Mathematics, Technical University of Munich, Munich, Germany; 7 Center for Biostructural Imaging of Neurodegeneration (BIN), University Medical Center Göttingen, Göttingen, Germany; 8 NanoTag Biotechnologies GmbH, Göttingen, Germany; 9 Department of Life Sciences, University of Trieste, Trieste, Italy

**Keywords:** cell biology, cell culture, microscopy, neuroscience

## Abstract

To unravel the complexity of biological processes, it is necessary to resolve the underlying protein organization down to single proteins. Here, we present a protocol for secondary label-based unlimited multiplexed DNA-PAINT (SUM-PAINT), a DNA-PAINT-based super-resolution microscopy technique that is capable of resolving virtually unlimited protein species with single-protein resolution. We describe the steps to prepare neuronal cultures, troubleshoot and conduct SUM-PAINT experiments, and analyze the resulting feature-rich neuronal cell atlases using unsupervised machine learning approaches.

For complete details on the use and execution of this protocol, please refer to Unterauer et al.^1^

## Highlights

• Neuron sample preparation for highly multiplexed super-resolution imaging
• SUM-PAINT spatial proteomic workflow, generating single-protein-resolved neuron atlases
• Multiplexed super-resolution data reconstruction and analysis with Picasso
• Classification of synaptic heterogeneity with machine-learning-based analysis

## Abstract (editor highlights)

Publisher’s note: Undertaking any experimental protocol requires adherence to local institutional guidelines for laboratory safety and ethics.

## Graphical abstract

**Figure:**
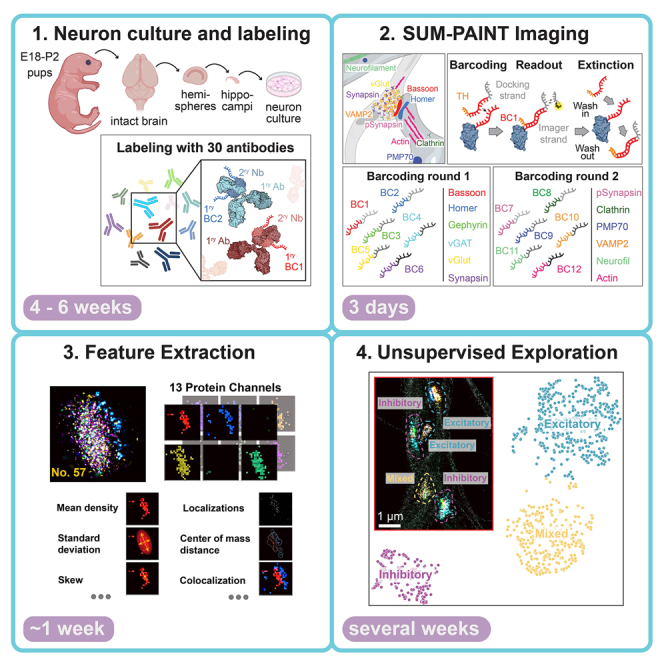

## Before you begin

In this protocol, we describe the details of obtaining neuronal architecture maps using SUM-PAINT super-resolution imaging. This technology was used to generate a 30 plex neuronal atlas in Unterauer et al.^1^ with the specific purpose of understanding the heterogeneity of synapses. It should be noted that SUM-PAINT multiplexing is not limited to neuronal imaging but can be applied to any target system of interest. Fluorescence imaging methods depend on available affinity reagents and before starting with SUM-PAINT, all affinity reagents (e.g., primary antibodies) need to be thoroughly tested in standard fluorescence microscopy. We will outline how to continue with troubleshooting these reagents, first for classical DNA-PAINT imaging and second for the assembly of multiplexing “codebooks” for SUM-PAINT. Following this assembly procedure, the codebooks in Unterauer et al.^1^ can be extended or modified to suit different application scenarios. We developed a comprehensive workflow from image acquisition to the analysis of morphometric features of synaptic structures.

### Institutional permissions

All animals were obtained from the University Medical Center Göttingen and were handled according to the specifications of the University of Göttingen and of the local authority, the State of Lower Saxony (Landesamt für Verbraucherschutz, LAVES, Braunschweig, Germany). Animal experiments were approved by the local authority, the Lower Saxony State Office for Consumer Protection and Food Safety (Niedersächsisches Landesamt für Verbraucherschutz und Lebensmittelsicherheit). If you wish to prepare primary neuronal cultures that are part of this protocol, ensure that you have all institutional permissions from the appropriate authorities for the use of the respected laboratory animals.

### Glia preparation (optional, required only for “sandwich” cultures; 1–3 weeks before neuronal culture)

**Timing: 2 days, ideally 3 weeks before neuron preparation for “sandwich” cultures**

1. One to three weeks before the neuron preparation grow glial cells in cell culture flasks.
2. Extract the cortices from six P0-P1 pups, as explained in primary neuron culture preparation section below, in dissection solution. Note that the same pups can be used for both glial preparation and mixed cultures, as it is possible to extract different brain regions and maximize culture yield.
3. Cut the cortices in small even size pieces using a scalpel.
4. Get the first 8 mL of dissection solution using a 10 mL pipette and transfer the tissue into a small falcon tube.
5. Wash the tissue by inverting the falcon tube 10 times and let the pieces sediment. Some of the tissue debris will be floating, but this will be discarded in the following steps.
6. Remove the supernatant and wash again with 8 mL of dissection solution.
7. Repeat the wash four times. At this point, floating tissue debris should be removed.
8. Remove the supernatant and add the prewarmed DNase I and Trypsin solutions to the dissection solution.
9. Invert the tube a couple of times and gently rotate it for 15 min at 37°C.
10. Take out the falcon and wait until there is a pellet. Remove the supernatant, leaving some solution inside, not to remove the pellet.
11. Wash the pellet four times with 8 mL of medium and carefully remove the supernatant.
12. During the last wash, collect all the solution and pipette it against the wall/bottom of a new falcon tube to homogenize the pellet.
13. Run the cell suspension through a 100 μm cell strainer on a 50 mL falcon tube and centrifuge at 800 rpm (150 g) for 10 min.
14. Remove the supernatant and resuspend it in 9 mL of glial medium without inducing bubbles.
15. Plate glia cells in three T75 cell culture flasks with perforated lid filled with 17 mL glial medium.
16. Next day: hit the flask gently to detach the microglia cells and fully exchange 20 mL glial medium per flask.

### Plating of the glia feeder layer (optional, just for “sandwich” cultures; 2–3 days before neuron plating)

**Timing: 1 day**

Note that glial cell preparation from P0-P2 rats should be performed 2–3 weeks prior to neuronal culture and is described in this protocol below. In case of mixed cultures, the feeder layer is not required, since the Neurobasal-A already promotes the survival and growth of a small population of glia that will mix with and support the neurons.

17. Coat the wells of 12-well plates with 0.1 mg/mL poly-*L*-lysine (PLL) in borate buffer.
18. Remove the PLL solution, wash 3 times with sterile double distilled water.
19. Add glial medium.
20. For glial seeding, remove the glial medium from the T75 cell culture flask.
21. Add 3 mL trypsin and incubate in the cell culture incubator at 37°C, 5% CO_2_ for ∼3 min.
22. Inactivate the trypsin with 6 mL of glial medium and collect the cell suspension for centrifugation.
23. Centrifuge at 800 rpm (150 g) for 9 min.
24. Remove the supernatant and resuspend the cells in fresh glial medium.
25. Count the cells using a Neubauer chamber or any other cell counting equipment.
26. Seed glial cells at 10,000-15,000 cells per well density three days prior neuron plating. The density of cells seeded depends on their age and proliferation ability.
27. For preconditioning, exchange the glial medium to N2-medium the night before the neuron preparation.

### Coverslip preparation

**Timing: 1 day**

28. Incubate the required number of 18 mm diameter coverslips (1.5#) in concentrated nitric acid for 24 h in ceramic racks in glass containers.

**CRITICAL:** Nitric acid is highly corrosive, handle under a chemical fume hood using appropriate personal protective equipment and dispose it according to the waste disposal regulations. When disposing use the appropriate glass containers and never mix with other substances as it might explode.

29. Wash the coverslips to remove the residual nitric acid with deionized water 5 times (for at least 5 min each) and sterilize the coverslips in the autoclave at 121°C for 30 min.
30. For “sandwich” cultures add four paraffin dots on each coverslip for further separation from glia feeder layer. To add the paraffin dots, melt the paraffin and use a glass Pasteur pipette to leave small drops on the coverslip.
31. Incubate the coverslips with 1 mg/mL PLL in borate buffer in the cell culture incubator at 37°C, 5% CO_2_ overnight (approximately 16 h), avoiding evaporation.
32. Remove the PLL solution, transfer the coverslips to 12-well plates, wash 3 times (each for at least 5 min) with sterile double distilled water.
33. Remove the water as much as possible by aspiration (but do not let it air dry).
34. Quickly add 1 mL of neuron plating medium per well on the day of primary neuron culture preparation.

***Note:*** PLL-coated coverslips (once dried) can be kept in 12-well plates for one week after closing them well with parafilm and keeping them at 4°C.

#### Primary neuron culture preparation

**Timing: 2–3 weeks depending on the required neuronal age**

**Figure 1:**
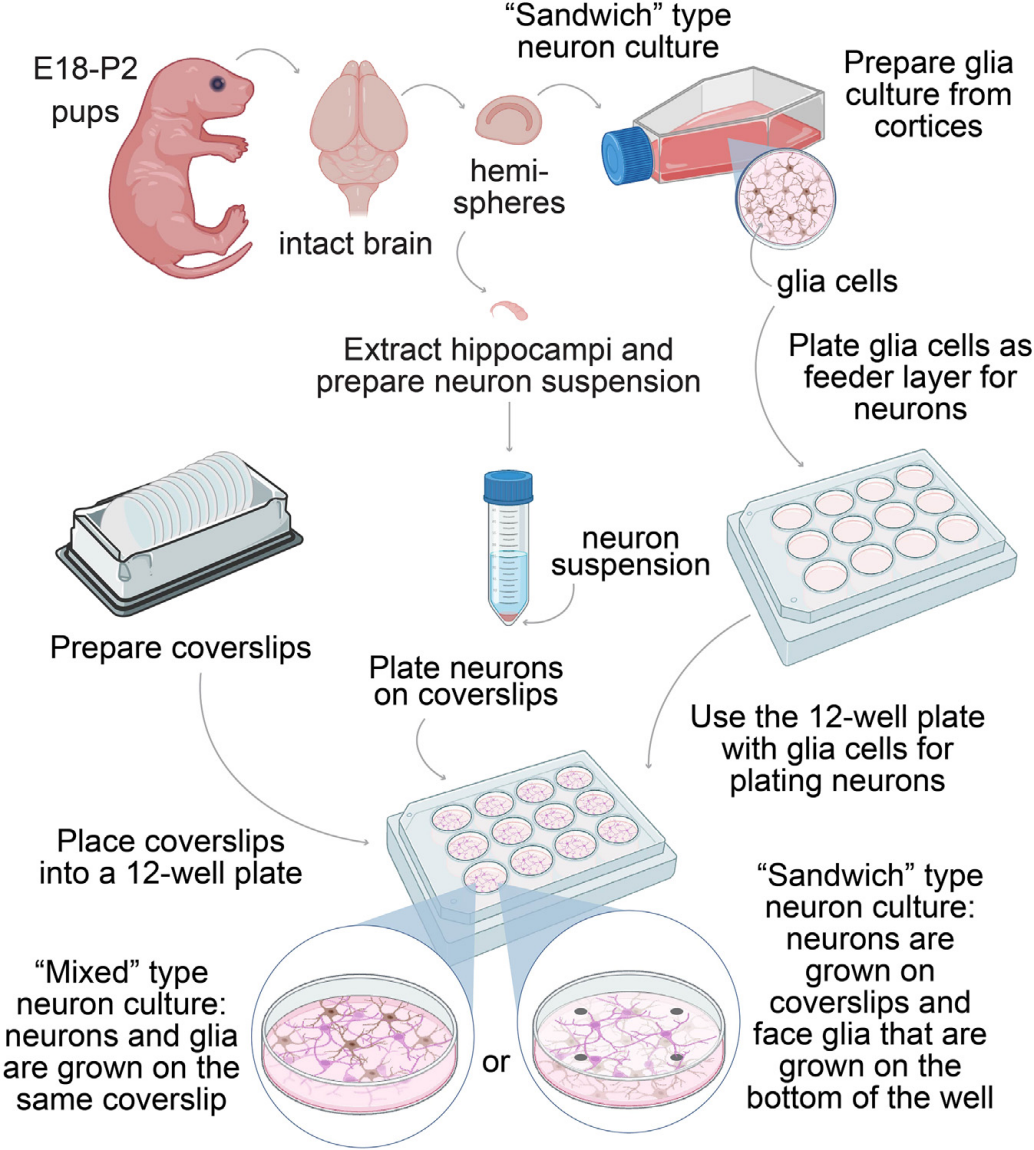
Workflow for preparation of neuronal primary cultures Steps describing neuron preparation workflow. Hemispheres are dissected and the hippocampi are isolated from E18 or P2 pups. Depending on “Mixed” type culture or “Sandwich” type culture neurons and glia are either grown on the same coverslip or neurons are grown on coverslips facing downwards with glia cells serving as a feeder layer.

35. To obtain primary neuronal culture (Figure 1), hippocampi have to be extracted from embryonic day 18 (E18; “sandwich” culture) or postnatal day 0-2 pups (P0-P2 “mixed” culture).^3^^,^^4^ The age difference in the case of E18 helps to reduce the number of glial precursors and ensures that the cultures have as little glial contamination as possible. P0-P2 cultures make these experiments more practical as it is not necessary to sacrifice the mother to extract the pups. The use of later ages (>P2) reduces the number of neurons that survive dissociation and is therefore avoided. Cultures should have an equal mix of either sex.

**CRITICAL:** If you need to prepare mixed cultures, 1–3 weeks before, the preparation of feeder layer cultures will need to be initiated (see below).

36. Decapitate E18 or P0-2 pups at the base of the skull and hold thin straight forceps (e.g., Dumont no. 7) in the orbits to immobilize the head.
37. Cut the skin along the sagittal suture from posterior to anterior side.
38. Cut the skull along the sagittal suture from posterior to anterior side and along the bone plate below the ears on both sides.
39. Open the skull using scissors and bent forceps.
40. Carefully remove the brain from the cranial cavity using a spatula and immerse it into precooled Hanks' Balanced Salt Solution (HBSS).
41. Once the brain is extracted, quickly proceed with dissection at the microscope.
42. Remove the meninges with forceps, make a cut along sagittal line using a scalpel and separate the two hemispheres.
43. Carefully remove the meninges, which can be identified by the presence of small blood vessels. Now the hippocampus should be exposed.
44. Remove the midbrain and separate the cortex with hippocampus using bent forceps (e.g., Dumont no. 7). To identify the hippocampus once the meninges have been removed, orient the hemisphere with the dorsal portion of the brain facing upward and identify the tissue fold. The flattened folded structure is the hippocampus. Refer to standard mouse brain atlases for detailed anatomical information.
45. Remove the hippocampus from both cortices using bent forceps. To remove the hippocampus, carefully insert bent forceps along the lateral edge of the cortex, gently retract to expose the hippocampus, then grip and lift it out by following its curvature. For pictures see Puppo et al.^5^
46. Once all required hippocampi are collected put them in a separate dish with precooled HBSS.
47. Chop the hippocampi with a razor blade in small 1 mm pieces.
48. Collect all pieces in a 15 mL falcon tube filled with 10 mL HBSS.
49. Let the bigger pieces sediment, remove the HBSS, replace with 10 mL fresh HBSS.
50. Let the pieces sediment, remove the HBSS, apply 10 mL of enzymatic solution pre-warmed to 37°C.
51. Gently rotate for 60 min at 37°C.
52. Let pieces sediment, remove the enzymatic solution, add 5 mL inactivation solution pre-warmed to 37°C.
53. Incubate for 5 min at 37°C.
54. Let the pieces sediment, remove the inactivation solution, add 10 mL of plating medium.
55. Let the pieces sediment, remove the plating medium, add 3 mL of plating medium and homogenize 10 times with a 10 mL cell culture pipette (limit the production of bubbles).
56. Filter the cell suspension through a 100 μm cell strainer on a 50 mL falcon tube.
57. Gently wash the cell strainer with 10 mL plating medium.
58. Centrifuge the cell suspension at 800 rpm (150 g) for 9 min.
59. Remove the supernatant and leave 1.5 mL inside the falcon tube.
60. Add 5 mL of fresh plating medium and resuspend the pellet with a 10 mL pipette.
61. Count the cells using a conventional cell counter. A Neubauer chamber or any other cell counter equipment can be used.
62. Seed cells on PLL-coated coverslips at 50,000 (“sandwich” culture) or 80,000 (“mixed” culture) cells per well density.
63. Replace plating medium with Neurobasal-A culture medium after 1–2 h for mixed cultures.
64. For the “sandwich” culture, 3–4 h after neuron plating, flip the coverslips with neurons to face the glia feeder layer.
65. Replace 1/5 of the medium from the wells with fresh N2-culture medium twice a week and grow neurons until required day *in vitro* (DIV), for example 14–22 DIV.
66. Limit the evaporation in the incubator opening it as little as possible and ensuring that it contains the correct amount of distilled water.

***Note:*** Growing neurons longer than 22 DIV is difficult and might result in compromised morphology and physiology.

67. For the “mixed” culture, add 10 μL of 8.1 mM 2′-deoxy-5-fluorodeoxyuridine stock in each well on the day of plating to inhibit glial proliferation.

***Note:*** Brains from each pup usually yield around 0.5–1 million neurons for mixed culture.

### Neuron fixation

**Timing: 35 min**

68. Fix the neurons at a required DIV by removing neuronal media and adding 1 mL prewarmed 4% paraformaldehyde solution per 12-well plate well for 30 min.

***Note:*** For “sandwich” culture move the coverslips neuron side up to a new 12-well plate which has been pre-filled with 1 mL of fixative in the required wells.

69. After fixation, remove the paraformaldehyde solution and rinse the neurons two times for 5 min with PBS.

**CRITICAL:** Paraformaldehyde is toxic. Handle under the fume hood and wear appropriate personal protective equipment and dispose it according to the waste disposal regulations.

## Key resources table

**Table:** 

REAGENT or RESOURCE	SOURCE	IDENTIFIER
**Antibodies**		

Mouse monoclonal anti-Bassoon (final dilution: 1:200)	Enzo	Cat#ADI-VAM-PS003-F; RRID:AB_11181058
Rabbit polyclonal anti-Homer 1 (final dilution: 1:200)	Rabbit polyclonal anti-Homer 1	Rabbit polyclonal anti-Homer 1
Mouse monoclonal anti-Gephyrin (final dilution: 1:200)	Synaptic Systems	Cat#147 011; RRID:AB_887717
Rabbit polyclonal anti-VGAT (final dilution: 1:200)	Invitrogen	Cat#PA5-27569; RRID:AB_2545045
Rabbit polyclonal anti-Phospho-Synapsin 1 (Ser549) (final dilution: 1:200)	Invitrogen	Cat#PA1-4697; RRID:AB_2175503
Rabbit monoclonal anti-Tom20 (final dilution: 1:100)	Abcam	Cat#ab186735; RRID:AB_2889972
Rabbit polyclonal anti-VGlut1 (final dilution: 1:200)	Synaptic Systems	Cat#135303; RRID:AB_887875
Mouse monoclonal anti-Neurofilament M (final dilution: 1:300)	Synaptic Systems	Cat#171231; RRID:AB_2814931
Rabbit polyclonal anti-Cav2.1 (final dilution: 1:150)	Synaptic Systems	Cat#152 203; RRID:AB_2619841
Mouse monoclonal anti-Rab3a (final dilution: 1:100)	Synaptic Systems	Cat#107111; RRIDAB_887770
Rabbit polyclonal anti-PMP70 (final dilution: 1:200)	Abcam	Cat#ab85550; RRID:AB_10672335
Mouse monoclonal anti-SV2B (final dilution: 1:200)	Synaptic Systems	Cat#119 111; RRID:AB_11042616
Mouse monoclonal anti-Vamp2 (final dilution: 1:300)	Synaptic Systems	Cat#104211; RRID:AB_887811
Mouse monoclonal anti-alpha synuclein (final dilution: 1:200)	Synaptic Systems	Cat#128 211; RRID:AB_2619811
Rabbit polyclonal anti-Synaptophysin (final dilution: 1:150)	Synaptic Systems	Cat#101 002; RRID:AB_887905
Rabbit polyclonal anti-Spinophilin (final dilution: 1:150)	Synaptic Systems	Cat#399003; RRID:AB_2721103
Mouse monoclonal anti-Neurofilament L (final dilution: 1:200)	Synaptic Systems	Cat#171011; RRID:AB_2891275
Rabbit polyclonal anti-Neurobeachin (final dilution: 1:200)	Synaptic Systems	Cat#194003; RRID:AB_2149112
Mouse monoclonal anti-βII Spectrin (final dilution: 1:100)	BD Biosciences	Cat#612562; RRID:AB_399853
Rabbit polyclonal anti-Synapsin 1/2 (final dilution: 1:200)	Synaptic Systems	Cat#106003; RRID:AB_2619773
Mouse monoclonal anti-Syntaxin6 (final dilution: 1:200)	BD Biosciences	Cat#610635; RRID:AB_397965
Rabbit polyclonal anti-alpha-Internexin (final dilution: 1:150)	LSBio	Cat#LS-C24890-50; RRID:AB_902082
Rabbit polyclonal anti-Clathrin heavy chain (final dilution: 1:200)	Abcam	Cat#ab21679; RRID:AB_2083165
Rabbit polyclonal anti-CamKII phosphor (final dilution: 1:300)	Rockland	Cat#612-401-C71; RRID:AB_11181161
sdAB anti-Synaptotagmin1 (final dilution: 1:300)	NanoTag Biotechnologies	Cat#N2302
Rabbit polyclonal anti-alpha-Tubulin (final dilution: 1:300)	Abcam	Cat#ab18251; RRID:AB_2210057
Rabbit recombinant anti-Tau (final dilution: 1:300)	Synaptic Systems	Cat#314008; RRID:AB_2891266
Mouse monoclonal anti-ac-tubulin (final dilution: 1:300)	Sigma	Cat#T7451; RRID:AB_609894
sdAB anti-PSD95 (final dilution: 1:300)	NanoTag Biotechnologies	Cat#N3705-250ug; RRID:AB_3076107
sdAB anti-rabbit IgG	NanoTag Biotechnologies	Cat#N2405-250ug; RRID:AB_3076046
sdAB anti-mouse Ig kappa light chain	NanoTag Biotechnologies	Cat#N1205-250ug; RRID:AB_3075962
FluoTag-X2 anti-Synaptotagmin 1 (sdAB) (final dilution: 1:300)	NanoTag Biotechnologies	Cat#N4302; RRID:AB_3076137
FluoTagX2 anti-VGlut1 (sdAB) (final dilution: 1:300)	NanoTag Biotechnologies	Cat#N1602; RRID:AB_3076006
Multiplexing blocker rabbit	NanoTag Biotechnologies	Cat#K0202-50; RRID:AB_3075894
Multiplexing blocker mouse	NanoTag Biotechnologies	Cat#K0102-50
Lifeact	MPI Biochemistry Core Facility	N/A

**Biological samples**		

Rat primary hippocampal neurons (Wistar albino [*Rattus Norvegicus*])	University Medical Center Göettingen	RGD_13508588

**Chemicals, peptides, and recombinant proteins**		

Sodium chloride (5 M)	Thermo Fisher Scientific	Cat#AM9759
UltraPure water	Thermo Fisher Scientific	Cat#10977-035
EDTA (0.5 M, pH 8.0)	Thermo Fisher Scientific	Cat#AM9260G
1× PBS pH 7.2	Thermo Fisher Scientific	Cat#20012-019
10× PBS	Thermo Fisher Scientific	Cat#70011051
Salmon sperm DNA	Thermo Fisher Scientific	Cat#15632011
Triton X-100	Carl Roth	Cat#6683.1
Paraformaldehyde aqueous solution (PFA 16%)	Electron Microscopy Sciences	Cat#15710
BSA	Sigma-Aldrich	Cat#A4503-10G
Tween 20	Sigma-Aldrich	Cat#P9416-50ML
Methanol	Sigma-Aldrich	Cat#32213-2.5L
(±)-6-hydroxy-2,5,7,8-tetra-methylchromane-2-carboxylic acid (Trolox)	Sigma-Aldrich	Cat#238813-5G
Sodium hydroxide (1 M)	VWR	Cat#31627.290
Protocatechuate 3,4-dioxygenase pseudomonas (PCD)	Sigma-Aldrich	Cat#P8279
3,4-dihydroxybenzoic acid (PCA)	Sigma-Aldrich	Cat#37580-25G-F
0.05% trypsin-EDTA	Thermo Fisher Scientific	Cat#25300-054
Tris (1 M)	Thermo Fisher Scientific	Cat#AM9855G
Dextran sulfate 50% solution	VWR	Cat#E516-100ML
Ethylene carbonate 98% solid	Sigma-Aldrich	Cat#E26258
20× SSC	Thermo Fisher Scientific	Cat#AM9763
Bifunctional maleimide-DBCO linker	Sigma-Aldrich	Cat#760668
Ammonium chloride	Merck	Cat#12125-02-9
Sodium azide (NaN_3_) 1% solution	G-Biosciences	Cat#786-750
Nitric acid 69%	Merck	Cat#1.01799
Sodium tetraborate decahydrate	Sigma	Cat#S9640
Boric acid	Sigma	Cat#B0252-500g
poly-*L*-lysine	Merck	Cat#P2658
Minimum essential media (MEM) no phenol red, no glutamine	Gibco	Cat#51200-046
Glucose	Merck	Cat#108342
Gibco penicillin-streptomycin (10,000 U/mL)	Thermo Fisher Scientific	Cat#15140-122
Normal horse serum	VWR International GmbH	Cat#S900-500
*L*-Glutamine	Thermo Fisher Scientific	Cat#25030-024
Dulbecco’s modified Eagle’s medium (DMEM)	Merck	Cat#D5671
*L*-Cysteine	Merck	Cat#30090
CaCl_2_	Merck	Cat#102382
Papain	Worthington Biochemical	Cat#LS003126
Fetal calf serum	Merck	Cat#S0615
Bovine serum albumin	AppliChem	Cat#A1391
Neurobasal-A	Thermo Fisher Scientific	Cat#10888-022
B27	Thermo Fisher Scientific	Cat#17504-044
GlutaMAX	Thermo Fisher Scientific	Cat#35050-038
N-2 supplement	Thermo Fisher Scientific	Cat#17502048
Sodium pyruvate	Thermo Fisher Scientific	Cat#11360-070
Calcium-, magnesium-, and bicarbonate-free Hank’s balanced salt solution (HBSS)	Thermo Fisher Scientific	Cat# 4175-053
N-(2-Hydroxyethyl)piperazine-N′-(2-ethanesulfonic acid) (HEPES)	Invitrogen	Cat#15630-056
DNase I	Sigma-Aldrich	Cat#10104159001
Gibco trypsin 2.5% no phenol red	Thermo Fisher Scientific	Cat#15090-046
2′-deoxy-5-fluorodeoxyuridine	Sigma	Cat#F0503
Uridine	Sigma	Cat#U3003
Paraformaldehyde	Merck	Cat#P6148
NaOH	Merck	Cat#1.06498.0500

**Oligonucleotides**		

R1-block: AGGAGGAGGAGGAGGAGGA	Metabion	N/A
R2-block: TGGTGGTGGTGGTGGTGGT	Metabion	N/A
R3-block: GAGAGAGAGAGAGAGAGAG	Metabion	N/A
R4-block: TGTGTGTGTGTGTGTGTGT	Metabion	N/A
R5-block: GAAGAAGAAGAAGAAGAAG	Metabion	N/A
R6-block: TTG TTG TTG TTG TTG TTG TT	Metabion	N/A
Primary barcode BC1: ATTAAC CGG TAG GCG ACG TT	Metabion	N/A
Primary barcode BC38: TATAACGGGTGAGCCTGCTG	Metabion	N/A
Primary barcode BC15: ACTATAACTCAGCTTAGCAT	Metabion	N/A
Primary barcode BC40: ATGAGGTCACTTATTCTCAA	Metabion	N/A
Primary barcode BC17: GCGCAACTTATTGCTATGCA	Metabion	N/A
Primary barcode BC30: TGGTAGCTGTGTACCGCAAA	Metabion	N/A
Primary barcode BC7: CGAAATTAGCGCGCGGTAAT	Metabion	N/A
Primary barcode BC20: TTGGTCCGGCGTAAACTATC	Metabion	N/A
Primary barcode BC27: GTCACAGGGTTCTACGCATG	Metabion	N/A
Primary barcode BC10: GTC ATA CCG TAT CGC CGA CT	Metabion	N/A
Primary barcode BC41: GAGTGAGTCATTCCTCTCAG	Metabion	N/A
Primary barcode BC43: ATCTAGTTCGTTCGGGCGTG	Metabion	N/A
Primary barcode BC25: ACAATACGCCCGTAGTACCA	Metabion	N/A
Primary barcode BC8: TAG ATC GCG ACT TTC GAG GT	Metabion	N/A
Primary barcode BC33: ACGTGTTAGTCGGGGTACAC	Metabion	N/A
Primary barcode BC34: GGTGGCATACTCCCTATTTT	Metabion	N/A
Primary barcode BC11: TAT TCG CGT TGG CCG CAA TT	Metabion	N/A
Primary barcode BC12: ATG AAT GGT CGT CGC GAA CC	Metabion	N/A
Primary barcode BC31: TTAGGTTCGTCCGATCTTCC	Metabion	N/A
Primary barcode BC32: GCTTATTTCGCCACGATTTA	Metabion	N/A
Primary barcode BC44: AAGTCGAACGGACCGTTTAT	Metabion	N/A
Primary barcode BC29: CTGTTGCTACGGACGTCTAC	Metabion	N/A
Primary barcode BC36: ACCTCACTAGTAAAACGAGC	Metabion	N/A
Primary barcode BC19: TTAGTCGGCGGTCGCATTTC	Metabion	N/A
Primary barcode BC45: TAAGATACGTCGACCCGATT	Metabion	N/A
Primary barcode BC22: GTCTTAAACATGCGAGAGCG	Metabion	N/A
Primary barcode BC5: ATT CAT ATC GCG ACG TAT AC	Metabion	N/A
Primary barcode BC42: CTGTCTACTACGAGTAAGCT	Metabion	N/A
Primary barcode BC46: TAAACCCGCGTACCTCGATT	Metabion	N/A
Secondary label sequence BC1: TCC TCC TCC TCC TCC TCC TAACGT CGCCTACCGGTTAATGGTCTTGTGG	Metabion	N/A
Secondary label sequence BC38: ACCACCACCACCACCACCA CAGCAG GCTCACCCGTTATA GGTCTTGTGG	Metabion	N/A
Secondary label sequence BC15: CTC TCT CTC TCT CTC CTC T ATGCT AAGCTGAGTTATAGT GGTCTTGTGG	Metabion	N/A
Secondary label sequence BC40: ACA CAC ACA CAC ACA ACA C TTGA GAATAAGTGACCTCAT GGTCTTGTGG	Metabion	N/A
Secondary label sequence BC17: CTTCTTCTTCTTCTTCTTC TGCATAGC AATAAGTTGCGC GGTCTTGTGG	Metabion	N/A
Secondary label sequence BC30: AACAACAACAACAACAACAA C TTTGCGG TACACAGCTACCA GGTCTTGTGG	Metabion	N/A
Secondary label sequence BC7: TCC TCC TCC TCC TCC TCC T ATT ACC GCG CGC TAA TTT CGG GTC TTG TGG	Metabion	N/A
Secondary label sequence BC20: ACCACCACCACCACCACCA GATAGTTTA CGCCGGACCAA GGTCTTGTGG	Metabion	N/A
Secondary label sequence BC27: CTC TCT CTC TCT CTC CTC T CATGCGTA GAACCCTGTGAC GGTCTTGTGG	Metabion	N/A
Secondary label sequence BC10: ACA CAC ACA CAC ACA ACA AGTCGGCG ATACGGTATGAC GGTCTTGTGG	Metabion	N/A
Secondary label sequence BC41: CTTCTTCTTCTTCTTCTTC CTGAGAGGAA TGACTCACTC GGTCTTGTGG	Metabion	N/A
Secondary label sequence BC43: AACAACAACAACAACAACAA C CACGCCC GAACGAACtAGAT GGTCTTGTGG	Metabion	N/A
Secondary label sequence BC25: TCCTCCTCCTCCTCCTCCT TGGTACTACG GGCGTATTGT GGTCTTGTGG	Metabion	N/A
Secondary label sequence BC8: ACCACCACCACCACCACCA ACCTCGAAA GTC GCGATCTA GGTCTTGTGG	Metabion	N/A
Secondary label sequence BC33: CTC TCT CTC TCT CTC CTC T GTGTACC CCGACTAACACGT GGTCTTGTGG	Metabion	N/A
Secondary label sequence BC34: ACA CAC ACA CAC ACA ACA C AAAATA GGGAGTATGCCACC GGTCTTGTGG	Metabion	N/A
Secondary label sequence BC11: CTTCTTCTTCTTCTTCTTC AATTGCGGCC AACGCGAATA GGTCTTGTGG	Metabion	N/A
Secondary label sequence BC12: AACAACAACAACAACAACAA C GGTTCG CGACGACCATTCAT GGTCTTGTGG	Metabion	N/A
Secondary label sequence BC31: TCC TCC TCC TCC TCC TCC T GGAAGAT CGGACGAACCTAA GGTCTTGTGG	Metabion	N/A
Secondary label sequence BC32: ACCACCACCACCACCACCA TAAATCGTG GCGAAATAAGC GGTCTTGTGG	Metabion	N/A
Secondary label sequence BC44: ACA CAC ACA CAC ACA ACA C ATAAAC GGTCCGTTCGACTT GGTCTTGTGG	Metabion	N/A
Secondary label sequence BC29: CTTCTTCTTCTTCTTCTTC GTAGACGTCC GTAGCAACAG GGTCTTGTGG	Metabion	N/A
Secondary label sequence BC36: AACAACAACAACAACAACAA C GCTCGT TTTACTAGTGAGGT GGTCTTGTGG	Metabion	N/A
Secondary label sequence BC19: TCC TCC TCC TCC TCC TCC T GAAATGC GACCGCCGACTAA GGTCTTGTGG	Metabion	N/A
Secondary label sequence BC45: CTC TCT CTC TCT CTC CTC T AATCGGG TCGACGTATCTTA GGTCTTGTGG	Metabion	N/A
Secondary label sequence BC22: ACA CAC ACA CAC ACA ACA C CGCTC TCGCATGTTTAAGAC GGTCTTGTGG	Metabion	N/A
Secondary label sequence BC5: CTTCTTCTTCTTCTTCTTC GTATACGTC GCGATATGAAT GGTCTTGTGG	Metabion	N/A
Secondary label sequence BC42: AACAACAACAACAACAACAA C AGCTTA CTCGTAGTAGACAG GGTCTTGTGG	Metabion	N/A
Secondary label sequence BC46: ACCACCACCACCACCACCA AATCGAGG TACGCGGGTTTA GGTCTTGTGG	Metabion	N/A
Toehold strand sequence BC1: CCACAAGACC ATTAACCGGTAGGCGACGTT	Metabion	N/A
Toehold strand sequence BC5: CCACAAGACC ATTCATATCGCGACGTATAC	Metabion	N/A
Toehold strand sequence BC7: CCACAAGACC CGAAATTAGCGCGCGGTAAT	Metabion	N/A
Toehold strand sequence BC8: CCACAAGACC TAGATCGCGACTTTCGAGGT	Metabion	N/A
Toehold strand sequence BC10: CCACAAGACC GTCATACCGTATCGCCGACT	Metabion	N/A
Toehold strand sequence BC11: CCACAAGACC TATTCGCGTTGGCCGCAATT	Metabion	N/A
Toehold strand sequence BC12: CCACAAGACC ATGAATGGTCGTCGCGAACC	Metabion	N/A
Toehold strand sequence BC15: CCACAAGACC ACTATAACTCAGCTTAGCAT	Metabion	N/A
Toehold strand sequence BC17: CCACAAGACCGCGCAACTTATTGCTATGCA	Metabion	N/A
Toehold strand sequence BC19: CCACAAGACC TTAGTCGGCGGTCGCATTTC	Metabion	N/A
Toehold strand sequence BC20: CCACAAGACC TTGGTCCGGCGTAAACTATC	Metabion	N/A
Toehold strand sequence BC22: CCACAAGACC GTCTTAAACATGCGAGAGCG	Metabion	N/A
Toehold strand sequence BC25: CCACAAGACC ACAATACGCCCGTAGTACCA	Metabion	N/A
Toehold strand sequence BC27: CCACAAGACC GTCACAGGGTTCTACGCATG	Metabion	N/A
Toehold strand sequence BC29: CCACAAGACC CTGTTGCTACGGACGTCTAC	Metabion	N/A
Toehold strand sequence BC30: CCACAAGACC TGGTAGCTGTGTACCGCAAA	Metabion	N/A
Toehold strand sequence BC31: CCACAAGACC TTAGGTTCGTCCGATCTTCC	Metabion	N/A
Toehold strand sequence BC32: CCACAAGACC GCTTATTTCGCCACGATTTA	Metabion	N/A
Toehold strand sequence BC33: CCACAAGACC ACGTGTTAGTCGGGGTACAC	Metabion	N/A
Toehold strand sequence BC34: CCACAAGACC GGTGGCATACTCCCTATTTT	Metabion	N/A
Toehold strand sequence BC36: CCACAAGACC ACCTCACTAGTAAAACGAGC	Metabion	N/A
Toehold strand sequence BC38: CCACAAGACC TATAACGGGTGAGCCTGCTG	Metabion	N/A
Toehold strand sequence BC40: CCACAAGACC ATGAGGTCACTTATTCTCAA	Metabion	N/A
Toehold strand sequence BC41: CCACAAGACC GAGTGAGTCATTCCTCTCAG	Metabion	N/A
Toehold strand sequence BC42: CCACAAGACC CTGTCTACTACGAGTAAGCT	Metabion	N/A
Toehold strand sequence BC43: CCACAAGACC ATCTAGTTCGTTCGGGCGTG	Metabion	N/A
Toehold strand sequence BC44: CCACAAGACC AAGTCGAACGGACCGTTTAT	Metabion	N/A
Toehold strand sequence BC45: CCACAAGACC TAAGATACGTCGACCCGATT	Metabion	N/A
Toehold strand sequence BC46: CCACAAGACC TAAACCCGCGTACCTCGATT	Metabion	N/A

**Software and algorithms**		

Custom analysis software	Unterauer et al., 2024^1^	Zenodo: https://doi.org/10.5281/zenodo.10212680
Picasso	Schnitzbauer et al., 2017^2^	Github: https://github.com/jungmannlab/picasso

**Other**		

No 1.5 glass slides 18 mm	Marienfeld	Cat#0112580
90 nm gold nanoparticles	Cytodiagnostics	Cat#G-90-100
TetraSpeck beads 0.1 μm	Thermo Fisher Scientific	Cat#T7279
Cell strainer 100 μm	Corning	Cat#352360
T25 cell culture flasks	Greiner	Cat#658 175
12-well cell culture plates	Th. Geyer	Cat#7696791
18 mm coverslips 1.5#	Th. Geyer LABSOLUTE	Cat#9161062
Inverted microscope body (ECLIPSE Ti2)	Nikon Instruments	MEA54000
Perfect Focus System	Nikon Instruments	MEP59394
Oil-immersion objective (Apo SRHP TIRF 100×, NA 1.49, oil)	Nikon Instruments	MRD01995
Immersion oil N 8 cc	Nikon Instruments	MXA22202
3D lens	Nikon Instruments	MED54301
Manual TIRF unit	Nikon Instruments	MEE59130
561 nm laser (1 W)	MPB Communications	N/A
Cleanup filter	Chroma Technology	ZET561/10
Beam splitter	Chroma Technology	ZT561rdc
Emission filter	Chroma Technology	ET600/50m
sCMOS camera (Zyla 4.2 plus)	Andor	N/A
2-component picodent glue eco-sil speed	picodent	1300 7100
2-component picodent glue eco-sil extrahart	picodent	1300 9100

## Materials and equipment

### Microscope setup

Imaging was carried out using an inverted microscope (equipped with a Perfect Focus System) using the objective-type TIRF configuration with an oil-immersion objective. A 561 nm laser, operated at a power density of 75 W/cm^2^, is used for excitation and is coupled into a single-mode fiber. The laser beam enters the microscope via a Nikon manual TIRF unit, passes through a cleanup filter and is directed into the objective via a fluorescence beam splitter. Fluorescence is spectrally filtered with an emission filter. An astigmatic lens is placed in the detection path to perform 3D imaging.^6^ Finally, fluorescence is imaged with an sCMOS camera without further magnification, resulting in an effective pixel size of 130 nm after 2 × 2 binning, leading to an imaging field of view of approximately 67 × 67 mm^2^. The camera readout sensitivity is set to 16-bit and the readout bandwidth to 200 MHz. Image acquisition and microscope control is performed using μManager.^7^

***Note:*** All buffer solutions during the neuron preparation are sterile filtered.

**Table:** Borate buffer (pH 8.5)

Reagent	Final concentration	Amount
Sodium tetraborate decahydrate	100 mM	2.012 g
Boric acid	100 mM	0.618 g
Double distilled water	-	100 mL
**Total**	**N/A**	**100 mL**

Bring to pH 8.5. Store at 4°C until use.

**Table:** poly-*L*-lysine (PLL) in borate buffer

Reagent	Final concentration	Amount
Borate buffer	1×	100 mL
poly-*L*-lysine	1 mg/mL	100 mg
**Total**	**N/A**	**100 mL**

Aliquot the solution and store at −20°C until use.

**Table:** Glial medium

Reagent	Final concentration	Amount
Minimum Essential Media (MEM)	1×	500 mL
glucose	0.5%	3 g
10.000 U/mL Penicillin/streptomycin	18 U/mL / 18 μg/mL	1 mL
200 mM *L*-Glutamine	1.8 mM	5 mL
Normal horse serum	9%	50 mL
**Total**	**N/A**	**556 mL**

The final solution can be warmed up to dissolve glucose and stored at 4°C.

**Table:** Neuron plating medium

Reagent	Final concentration	Amount
MEM	1×	100 mL
Glucose	3 mM	60 mg
Normal Horse Serum	9%	10 mL
200 mM *L*-Glutamine	1.8 mM	1 mL
**Total**	**N/A**	**111 mL**

The final solution can be warmed up to dissolve glucose and stored at 4°C.

**Table:** Enzymatic solution for neuron preparation

Reagent	Final concentration	Amount
Dulbecco’s MEM (DMEM)	1×	10 mL
*L*-cysteine	1.6 mM	2 mg
1 M CaCl_2_	1 mM	0.1 mL
0.5 M ethylenediaminetetraacetic acid (EDTA)	0.5 mM	0.1 mL
Papain	25 U/mL	150 μL
**Total**	**N/A**	**10.25 mL**

Add fresh papain every time, don’t freeze. Equilibrate the final solution with carbogen for 10–20 min, sterile filter and keep at 37°C during preparation until use.

**Table:** Inactivation solution for neuron preparation

Reagent	Final concentration	Amount
Fetal calf serum	10%	10 mL
Bovine serum albumin	5 mg/mL	50 mg
**Total**	**N/A**	**10 mL**

Fetal calf serum can be aliquoted and stored at −20°C. Store bovine serum albumin at 4°C. Prepare inactivation solution fresh every time.

**Table:** Neurobasal-A neuron culture medium

Reagent	Final concentration	Amount
Neurobasal-A medium	1×	500 mL
50× B27	1×	10 mL
200 mM GlutaMAX	2 mM	5 mL
**Total**	**N/A**	**515 mL**

Store at 4°C until use.

**Table:** N2 culture medium

Reagent	Final concentration	Amount
MEM	1×	500 mL
N2 Supplement (100×)	1×	5 mL
L-Glutamine (200 mM)	2 mM	5 mL
Sodium Pyruvate (100 mM)	1 mM	5 mL
Glucose	0.6%	3 g
**Total**	**N/A**	**515 mL**

Store at 4°C until use.

**Table:** Glia dissection solution

Reagent	Final concentration	Amount
Calcium, Magnesium and Bicarbonate-free Hanks’ Buffered Salt Solution (HBSS)	1×	500 mL
1 M N-(2-Hydroxyethyl)piperazine-N′-(2-ethanesulfonic acid) (HEPES)	9.8 mM	5 mL
Trypsin 2.5%	0.75%	1.5 mL
10 mg/mL DNase I	3%	1.5 mL
**Total**	**N/A**	**8.5 mL**

Glia dissection solution without enzymes can be stored at 4°C until use. Enzyme solutions are added during the preparation (step 8 in Glia preparation for “sandwich” cultures)

**Table:** 2′-deoxy-5-fluorodeoxyuridine (FUDR) solution

Reagent	Final concentration	Amount
DMEM	1×	12.5 mL
2′-deoxy-5-fluorodeoxyuridine	8.1 mM	25 mg
Uridine	20.4 mM	62.5 mg
**Total**	**N/A**	**12.5 mL**

Mix thoroughly and sterile filter the final solution. Aliquots can be stored at −20°C until use.

**Table:** 4% paraformaldehyde solution

Reagent	Final concentration	Amount
Paraformaldehyde (PFA)	4%	20 g
Double distilled water	-	450 mL
10× PBS	1×	50 mL
1 M NaOH	-	A few drops
**Total**	**N/A**	**500 mL**

***Note:*** Paraformaldehyde (PFA) is available in pre-prepared aqueous solutions (e.g. PFA 16%) to minimize risks associated with handling hazardous powder. However, this does not eliminate exposure risks, as PFA solutions can still release formaldehyde gas.

**Preparation**: Add PFA to double distilled water first, stir at ∼55°C–60°C until dissolved. A few drops of 1 M NaOH help to dissolve the PFA. Then add the remaining volume of 10× PBS. Adjust pH to 7-8. Aliquots can be stored at −20°C until use.

**CRITICAL:** Paraformaldehyde is a hazardous chemical when inhaled or in contact with skin. Handle under the fume hood with appropriate personal protective equipment (gloves, lab coat and protective glasses).

**Table:** Buffer C+

Reagent	Final concentration	Amount
EDTA (0.5 M)	0.1 mM	10 μL
NaCl (5 M)	500 mM	5 mL
Tween-20	0.05% (v/v)	25 μL
1× PBS	N/A	44.965 mL
**Total**	**N/A**	**50 mL**

Store at room temperature for up to 1 month.

**Table:** Buffer C washing buffer (C_w_)

Reagent	Final concentration	Amount
NaCl (5 M)	500 mM	5 mL
1× PBS	N/A	44.965 mL
**Total**	**N/A**	**50 mL**

Store at room temperature for up to 1 month.

**Table:** Antibody incubation buffer

Reagent	Final concentration	Amount
BSA	2% (wt/v)	1 g
EDTA (0.5 M)	1 mM	100 μL
Tween 20 (100%)	0.02% (v/v)	10 μL
NaN_3_ (10%)	0.05% (v/v)	250 μL
Salmon-sperm DNA (10 mg/mL)	0.05 mg/mL	250 μL
1× PBS	N/A	49.39 mL
**Total**	**N/A**	**50 mL**

Store at 4°C for up to 3 months.

**CRITICAL:** Sodium azide (NaN_3_) is toxic when inhaled or in contact with skin. Handle under the fume hood with appropriate personal protective equipment (gloves, lab coat and protective glasses).

**Table:** 0.5% Triton solution

Reagent	Final concentration	Amount
Triton X-100	0.5% (v/v)	75 μL
1× PBS	N/A	14.925 mL
**Total**	**N/A**	**15 mL**

Store at room temperature for up to 3 months.

**Table:** 3% BSA solution

Reagent	Final concentration	Amount
BSA	3% (wt/v)	1 g
1× PBS	N/A	50 mL
**Total**	**N/A**	**50 mL**

Store at 4°C for up to 3 months.

**Table:** Post-fixation buffer

Reagent	Final concentration	Amount
Paraformaldehyde aqueous solution (PFA 16%)	4% (v/v)	125 μL
1× PBS	N/A	375 μL
**Total**	**N/A**	**500 μL**

Make fresh for each coverslip.

**CRITICAL:** Paraformaldehyde is toxic when inhaled or in contact with skin. Handle under the fume hood with appropriate personal protective equipment (gloves, lab coat and protective glasses).

**Table:** Permeabilization buffer

Reagent	Final concentration	Amount
0.5% Triton solution	0.25% (v/v)	250 μL
1× PBS	N/A	250 μL
**Total**	**N/A**	**500 μL**

Make fresh for each coverslip.

**Table:** Blocking buffer

Reagent	Final concentration	Amount
Salmon sperm DNA (10 mg/mL)	0.05 mg/mL	1.5 μL
3% BSA solution	N/A	298.5 μL
**Total**	**N/A**	**300 μL**

Make fresh for each coverslip.

**Table:** Combined permeabilization/blocking buffer

Reagent	Final concentration	Amount
0.5% Triton solution	0.25% (v/v)	150 μL
Salmon sperm DNA (10 mg/mL)	0.05 mg/ML	1.5 μL
3% BSA solution	3% (wt/v)	148.5 μL
**Total**	**N/A**	**300 μL**

Make fresh for each coverslip.

**Table:** 2× SSC washing buffer

Reagent	Final concentration	Amount
20× SSC	N/A	5 mL
MiliQ water	N/A	45 mL
**Total**	**N/A**	**50 mL**

Store at room temperature for up to 3 months.

**Table:** Optimized hybridization buffer

Reagent	Final concentration	Amount
Dextran sulfate (50%)	10% (v/v)	5 mL
Ethylene carbonate (98%)	10% (v/v)	5 mL
20× SSC	4× SSC	5 mL
Tween-20	0.4% (v/v)	200 μL
Ultra-pure water	N/A	49.8 mL
**Total**	**N/A**	**50 mL**

Store at room temperature for up to 3 months.

**CRITICAL:** Ethylene carbonate is a hazardous chemical when inhaled or in contact with skin. Handle under the fume hood with appropriate personal protective equipment (gloves, lab coat and protective glasses).

**Preparation:** Liquefy ethylene carbonate in a preheated water bath (∼90°C) for buffer preparation.

**Table:** PCA 40× stock solution

Reagent	Final concentration	Amount
PCA	N/A	154 mg
NaOH (1 M)	N/A	approximately 1 mL
Ultra-pure water	N/A	10 mL
**Total**	**N/A**	**11 mL**

Store at −20°C in aliquots of 50 μL for up to 3 months.

**Preparation:** Prepare 154 mg PCA in 10 mL ultra-pure water (note that PCA will not yet dissolve, pH at around 2). In steps of 100 μL add 1 M NaOH until a pH 9.0 is achieved, note that PCA will fully dissolve (total volume of NaOH need ∼1 mL)

**Table:** PCD buffer

Reagent	Final concentration	Amount
KCl (1 M)	50 mM	1.5 mL
EDTA (0.5 M)	1 mM	60 μL
Tris-HCl (1 M, pH 8)	100 mM	3 mL
Glycerol	50% (v/v)	15 mL
Ultra-pure water	N/A	10.44 mL
**Total**	**N/A**	**30 mL**

Store at room temperature for up to 6 months.

**Table:** PCD 100× stock solution

Reagent	Final concentration	Amount
PCD	0.699 mg/mL	9.3 mg
PCD buffer	N/A	13.3 mL
**Total**	**N/A**	**13.3 mL**

Store at −20°C in aliquots of 20 μL for up to 3 months.

**Table:** Trolox 100× stock solution

Reagent	Final concentration	Amount
Trolox	N/A	100 mg
Methanol (100%)	N/A	430 μL
NaOH (1 M)	N/A	approximately 480 μL
Ultra-pure water	N/A	3.2 mL
**Total**	**N/A**	**4 mL**

Store at −20°C in aliquots of 20 μL for up to 3 months.

**Preparation:** Prepare 100 mg of Trolox and add 430 μL 100% methanol, mix vigorously by vortexing. Add 3.2 mL ultra-pure water to the solution (note that Trolox will not yet dissolve). At first add 350 μL 1 M NaOH and vortex vigorously, notice that Trolox will start dissolving. Add more of 1 M NaOH in steps of 20 μL and mix vigorously until Trolox is completely dissolved (total volume of NaOH ∼480 μL).

**Table:** Imaging buffer

Reagent	Final concentration	Amount
PCA 40× stock solution	1×	25 μL
PCD 100× stock solution	1×	10 μL
Trolox 100× stock solution	1×	10 μL
Buffer C+	N/A	950 μL
**Total**	**N/A**	**1 mL**

Make fresh for before each imaging round. 1 mL imaging buffer serves for 2 coverslips.

## Step-by-step method details

### Sample preparation for DNA-PAINT imaging

**Timing: ∼5 h**

**Figure 2:**
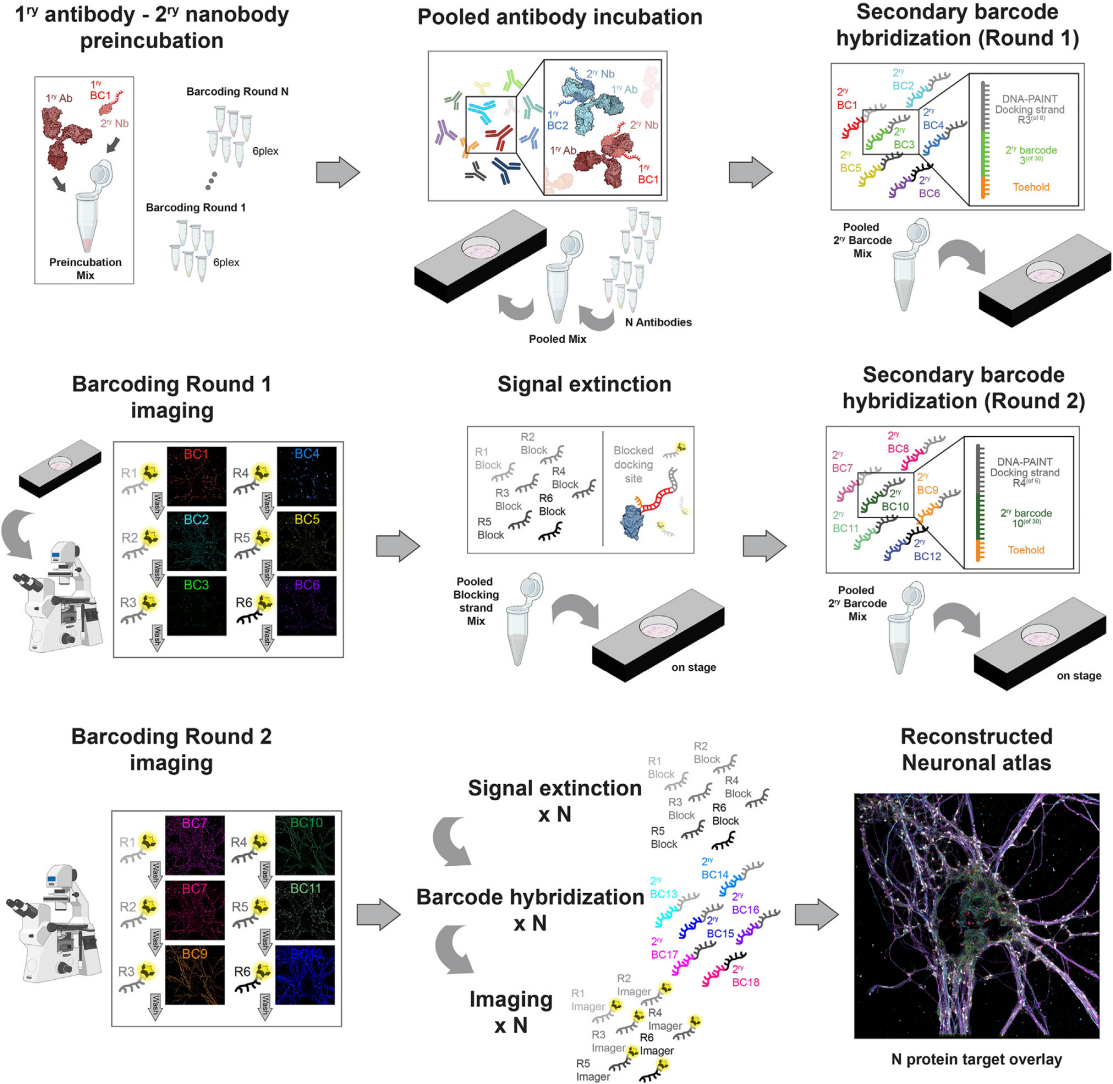
Workflow for SUM-PAINT sample preparation and imaging Steps describing labeling of the sample and SUM-PAINT labeling and imaging workflow. After primary and secondary antibody and nanobodies are preincubated in sets of 6 targets the pooled antibodies are incubated onto the sample. With secondary hybridization of barcoding round 1 the imaging can be conducted in sequential fashion using speed-optimized R-sequences. When the imaging is done signal extinction is done via blocking of the docking strands by the hybridization of a full complement. Once this step is completed the next barcoding round can start until all targets are acquired and the neuronal atlas can be reconstructed.

This section explains how to prepare neuron samples for immunolabeling, DNA-PAINT and SUM-PAINT imaging (Figure 2). In Unterauer et al.,^1^ mainly primary antibodies in combination with secondary nanobodies were used. The first staining round used 6 to 12 different combinations of primary antibodies and secondary nanobodies. In order to extend to more than 12 targets please refer to the next section 30-plex SUM-PAINT image acquisition.

1. Assemble 18 mm coverslip in custom-made sample chamber (see Figure S1).
  a. Take coverslip out of 12 well plate using fine precision tweezers and position it onto a small holder with neurons facing upwards.
  b. Add 10 μL of 1× PBS on top to keep coverslip humid.
  c. Prepare two-component picodent glue with 1:1 ratio and mix thoroughly.
  d. Distribute mixed picodent glue onto the outer edge of the sample chamber using a 10 μL pipette tip and gently press coverslip on, cells facing downward.
    ***Note:*** Picodent glue solidifies within a few minutes. SUM-PAINT acquisitions can take several days, and thus, it is crucial for the chamber not to leak during the acquisition process. Work as fast and precisely as possible when gluing the coverslip into the chamber.
    ***Note:*** Details for construction and sample preparation with the custom-made sample chamber can be seen in Figure S1. In theory any chamber able to immobilize an 18 mm coverslip, while allowing for buffer exchange, can be used.
  e. Wait up to 10 min for glue to dry and add 1× PBS into the sample chamber
    ***Note:*** Picodent offers different component glues with shorter and longer solidifying times. If sample leakage is a problem, use picodent eco-sil extrahart.
2. Aspirate 1× PBS and add 300 μL combined permeabilization/blocking buffer for permeabilization and blocking. Let the solution incubate on the sample for 45 min at RT (approximately 21°C).

***Note:*** Permeabilization and blocking can be separated into two steps. Here this would result in 0.25% Triton X-100 in PBS incubation for 20 min, followed 3 times washing with PBS and then blocking with 3% BSA + 0.05 mg/mL salmon spear DNA for 45 min.

***Note:*** Permeabilization and blocking times and concentrations of detergents may vary depending on the protein targets. 0.25% Triton X-100 was found to be the best concentration for the 30 targets in Unterauer et al.^1^ Before starting with SUM-PAINT, targets need to be evaluated in single target DNA-PAINT measurement to determine the best conditions for permeabilization.

3. Wash the sample three times with 1× PBS and add gold nanoparticles diluted 1:3 in PBS to it for 5 min at RT (approximately 21°C), followed by 3× washing with 1× PBS.

***Note:*** If gold nanoparticles seem to accumulate in the resulting images, vortexing the diluted solution beforehand is recommended.

**Pause point:** Samples can be stored for several days before immunolabeling; however, to ensure optimal sample quality, we recommend limiting storage to no longer than overnight (approximately 16 h).

4. Prepare premixed incubation stocks for primary antibodies and secondary nanobodies for the first barcoding round (6 targets).
  a. Pipette 10 μL of antibody incubation buffer in a 500 μL Eppendorf tube, for each target respectively.
  b. Add respective primary antibody in final dilution. For example, for Bassoon a final dilution of 1:200 is used. Having a total incubation volume of 300 μL for the sample chamber, 1.5 μL of the Bassoon Ab is added to the 10 μL preincubation volume (Refer to key resources table for primary antibody dilution and concentration information for all 30 targets).
  c. Add the respective secondary nanobody in the final concentration. For Bassoon the secondary nanobody with Barcode (BC) 1 was used at a dilution of 1:200 resulting in 1.5 μL. (Refer to key resources table for secondary nanobody dilution and concentration information for all 30 targets).
  d. Gently mix the preincubated volumes e.g., by flicking the tube, before quickly spinning it down using a mini table centrifuge (in total there should be six preincubation mixes prepared) and letting the solution incubate for at least 1 h at RT (approximately 21°C).
    ***Note:*** We found that an incubation time ranging from 1 h to overnight (approximately 16 h) incubation show similar results. For overnight incubation we recommend incubating at 4°C.
  e. Prepare blocking nanobody dilution by adding 1 μL of respective blocking nanobody (mouse kLC/rabbit at stock concentration of 30 μM) to 9 μL of 1× PBS (final concentration of 3 μM).
  f. Add 1 μL of matching species blocking nanobody to the respective primary antibody/secondary nanobody preincubation tubes (for Bassoon with a mouse kLC secondary nanobody the respective blocking nanobody would be unlabeled mouse kLC) and let the solution incubate for 5 min at RT (approximately 21°C).
    ***Note:*** In Unterauer et al.^1^ blocking nanobodies were ordered as unlabeled secondary nanobodies. NanoTag Biotechnologies now offers a multiplexing blocker kit, which was not evaluated in this study.
  g. Prepare final incubation by mixing 200 μL of antibody incubation buffer with 0.5 μL of each species blocking nanobody stock concentration (30 μM).
  h. Pool volume from preincubated primary antibody/ secondary nanobody tubes into the final incubation mix.
    ***Note:*** The incubation mix can be prepared for more than 6 targets. For the 30 plex imaging in Unterauer et al.^1^ six targets per barcoding round were prepared. For the 12 plex speed demonstration the incubation mix was prepared for 12 targets. When optimizing the primary antibody to secondary nanobody ratios, 12 or even more targets can be incubated at once. But moving to higher-plex experiments like in the 30 plex, it is highly recommended to split the incubation mixes.
5. Wash the sample with 1× PBS and apply the final incubation mix onto the sample for 90 min at RT (approximately 21°C).

***Note:*** Incubation times longer than 3 h can lead to cross-talk between the primary antibody/secondary nanobody pairs. Even though this cross-talk percentage remains low even after overnight (approximately 16 h) incubation (about 10%), it is highly recommended not to incubate longer than 2 h.

6. Wash sample 4× with 1× PBS, once with Buffer C_w_ and once with 2× SSC washing Buffer.
7. Prepare secondary-label incubation mix.
  a. Pipette 1 mL of Optimized hybridization buffer in a 1.5 mL Eppendorf tube.
    ***Note:*** Since the optimized hybridization buffer contains 10% dextran sulfate the viscosity of the buffer is relatively high. Be careful when pipetting to not introduce air bubbles in the volume.
  b. Add 1 μL of each secondary-label (stock concentration 100 μM) to a final concentration of 100 nM per label and total secondary-label barcode mix of 600 nM (for six strands).
    ***Note:*** Be careful when pipetting small volumes into optimized hybridization buffer. It is recommended to mix the solution gently after each pipetting step.
8. Apply the secondary-label incubation mix onto the sample for 15 min.
9. Wash the sample 5× with 2× SSC buffer and once with Buffer C_w_. Proceed to SUM-PAINT imaging within the next 30 min.

### 30-plex SUM-PAINT image acquisition

**Timing: Approximately 17 min per target plus 40 min extra for each additional barcoding round. For 30 targets approximately 30 h are needed.**

This section describes the detailed steps to acquire highly multiplexed images with SUM-PAINT (Figure 2). In Unterauer et al.,^1^ 30 targets were acquired, separating the antibody incubation steps to the beginning of each barcoding round. When imaging fewer targets (12 plex for example) these steps can be neglected, and the incubation can be done in a one-pot reaction as described in the “sample preparation for SUM-PAINT imaging” step. In Unterauer et al.,^1^ a specific antibody list with compatibility for DNA-PAINT and SUM-PAINT imaging was evaluated and any target on that list can be imaged with the same steps independent of the specific 30 plex codebook design. While this protocol is robust, imaging conditions should be adjusted depending on the sample condition and target requirements (please see troubleshooting section).

10. Prepare imaging solution.
  a. Pipette 950 μL of buffer C+ in a 1.5 mL Eppendorf tube.
  b. Add 10 μL of 100× PCD solution, 10 μL 100× Trolox solution and 25 μL 40× PCA solution
  c. Add the respective imager strand in a pre-evaluated concentration (as an example for Bassoon imaging in Unterauer et al.^1^ 100 pM of R1 imager were used).

***Note:*** Imager concentration depends strongly on the protein target in question. Blinking events should not show substantial overlap in every camera frame to achieve the highest localization precision. The conditions of optimal sampling and highest localization precision have been described in several studies, among them Schnitzbauer et al.^2^ and Strauss et al.^8^ DNA-PAINT imager concentration can be easily adjusted by preparing a new solution and simply exchanging the solution onto the sample until the respective image fulfills the requirements of the measurement regarding acquisition speed, sampling and localization precision

11. Wash the sample with 500 μL imaging solution and then apply 500 μL imaging solution onto the sample.
12. Clean the bottom of the coverslip with a methanol-dipped Kim wipe before going to the microscope.
13. Take DNA-PAINT images with a 100× objective (NA 1.49, e.g., Apo SR TIRF from Nikon Instruments).
14. Apply a single drop of silicone immersion oil to the center of the objective (make sure no air bubbles are in the oil droplet).
15. Mount the sample chamber onto the scope.

***Note:*** It is recommended to use double-sided sticky tape to fix the sample holder onto the stage, ensuring the sample is not moving during imaging and buffer exchange steps.

16. Find the optimal region of interest and imaging plane. It is recommended to use a higher imager concentration for one specific target of importance in a pre-screening approach. A Highly overlapping DNA-PAINT signal will appear almost similar to diffraction-limited imaging and the optimal z-plane and illumination angle can be determined. Unterauer et al.^1^ used VGAT as a specific pre-screening target, applying 200 pM of imager solution.

***Note:*** Finding the optimal region and plane for image acquisition can take several minutes to even hours. While not used in Unterauer et al.,^1^ using a diffraction-limited counter-staining in a separate excitation/detection channel is highly recommended to make this process easier. For neurons, a VGlut1 or Synaptotagmin nanobody (NanoTag Biotechnology) coupled to, for example, Alexa Fluor 488 or Alexa Fluor 647 can be added to the incubation mix and later used for this purpose. Note that the protein used for counterstaining can no longer be used in the SUM-PAINT multiplexed imaging.

17. Perform sequential imaging of barcoding round 1. In Unterauer et al.^1^ all imagers were coupled to Cy3B and are excited using a 560 nm laser.
  a. Set the laser power to approximately 15 mW (75 W/cm^2^) (laser power was measured out of the objective) and the exposure time to 100 ms.
  b. Set the acquisition conditions to 10,000 frames resulting in approximately 17 min per acquisition of a single target.
    ***Note:*** The number of frames to take depend on the target density, the acquisition time and the desired resolution. For example, by imaging with more frames and a lower imager concentration the signal can be further separated among the frames and the imaging result will have a higher localization precision. We recommend to use 10,000 to 15,000 frames for targets in the 30 plex imaging list in Unterauer et al.^1^ This gives a good compromise on imaging time and resulting resolution while ensuring high sampling of the target structure.
  c. Insert cylindrical lens for 3D acquisition and acquire image stack.
    ***Note:*** 3D acquisition via astigmatism, using a cylindrical lens, require a calibration measurement typically done with Tetraspeck beads immobilized on a glass coverslip (applying a solution of 1:200 dilution of beads in 5 M Tris onto a coverslip is enough to immobilize them). ‘Picasso: Localize’ offers an adapted version of Huang et al.^6^ described in the Picasso software package^2^ on GitHub to calibrate a z-stack of a Tetraspeck bead sample.
  d. After the first target is imaged wash the sample 4× with C_w_ buffer and introduce the imager solution for the next target (In Unterauer et al.^1^) the second target was Homer1 imaged with 150 pM and R2 imager).
  e. Continue this procedure until all six targets of barcoding round 1 are imaged.
18. After barcoding round 1, wash 3× with 1× PBS and apply nanobody blocking solution, consisting of 0.5 μL of each blocking nanobody species in 400 μL PBS, for 5 min onto the sample. In Unterauer et al.^1^) 0.5 μL of mouse kLC and 0.5 μL of rabbit IgG of a stock concentration of 30 μM were used.
19. Prepare the second nanobody incubation mix according to Step 4.

***Note:*** In order to save time, the incubation mix for the next barcoding should optimally be prepared beforehand. In Unterauer et al.,^1^ the incubation mix for barcoding round 2 was prepared while the incubation mix for barcoding round 1 was incubating onto the sample.

20. Apply the incubation mix for barcoding round 2 onto the sample for 90 min on stage at RT (approximately 21°C).
21. Wash the sample 4× with 1× PBS, once with buffer C_w_ and once with 2× SSC buffer
22. Prepare silencing mix for barcoding round 1.
  a. Pipette 1 mL of Optimized hybridization buffer in a 1.5 mL Eppendorf tube.
    ***Note:*** Since the Optimized hybridization buffer contains 10% dextran sulfate the viscosity of the buffer is relatively high. Be careful when pipetting to not introduce air bubbles in the volume.
  b. Add 1 μL of each blocking strand (stock concentration 100 μM) to a final concentration of 100 nM per label and total blocking strand mix of 600 nM (for six strands).
  c. Optionally add 1 μL of each toehold displacement-strand (stock concentration 100 μM) to a final concentration of 100 nM per label and a 600 nM toehold mediated strand-displacement mix.
    ***Note:*** Be careful when pipetting small volumes into optimized hybridization buffer. It is recommended to mix the solution gently after each pipetting step.
23. Apply the silencing solution for barcoding round 1 for 15 min on stage at RT (approximately 21°C).
24. Wash the sample 6× with 2× SSC buffer.
25. Prepare secondary-label mix for barcoding round 2 according to Step 7.
26. Apply the secondary-label mix for barcoding round 2 for 15 min on stage at RT (approximately 21°C).
27. Wash the sample 5× with 2× SSC buffer and once with Buffer C_w_.
28. Prepare imaging solution for the first target of barcoding round 2 according to Step 10 and start image acquisition of barcoding round 2 according to Step 17.
29. After barcoding round 2 the same procedure consisting of nanobody blocking according to Step 18, antibody/nanobody incubation (incubation mix preparation according to Step 4), silencing of the prior barcoding round (silencing mix preparation according to Step 22), hybridization of the next secondary-label barcoding round (secondary-label mix preparation according to Step 7) and image acquisition according to Step 17 can be repeated until all barcoding rounds and therefore all protein targets are imaged.

***Note:*** In Unterauer et al.^1^ 30 protein targets in 5 barcoding rounds were imaged. This procedure does not have to be done consecutively. After the image acquisition of one barcoding round is finished, the sample can be washed 3× with PBS and left in PBS for several hours (or overnight (approximately 16 h)) and the procedure for incubating the next barcoding round can be continued later/the next day. Nevertheless, it is not recommended to let the sample stay at RT (approximately 21°C) on stage for more than three days.

***Note:*** In Unterauer et al.^1^ the last protein target was actin, which was imaged using Lifeact according to Riedl et al.^9^ For imaging Lifeact a higher laser power and a shorter integration time is recommended, e.g. 25 mW laser power and 50 ms integration time.

### Super-resolved image reconstruction and neuronal atlas alignment

**Timing: ∼3 h**

This section outlines the steps for optimal DNA-PAINT and SUM-PAINT image reconstruction and alignment. In Unterauer et al.,^1^ the software package Picasso was used to localize individual molecules, “undrift” the localizations to reconstruct the super-resolved image of 30 neuron targets and align these targets into a neuronal atlas. The general steps on how to operate Picasso are described in Schnitzbauer et al.^2^ In theory any other software package for analysis of localization-based super resolution data can be employed, e.g., SMAP^10^ or microscope manufacturer provided software.

30. Fitting the photon distributions of molecular blinking with ‘Picasso: Localize’.
  a. Start ‘Picasso: Localize’ and open the movie stack (10,000 frames) of one protein target.
    ***Note:*** ‘Picasso: Localize’ indicates if the entire 10,000 frames are loaded or only a subset. The total number of frames as well as the current frame can be seen in the bottom right corner.
  b. Go to the button “Analyze” and then to the button “Parameters” to adjust the threshold for single-molecule detection. In Unterauer et al.,^1^ a net gradient of 5,000 and a box size (in camera pixel) of 9 was chosen.
    ***Note:*** The optimal net gradient and box size are strongly dependent on the imaging conditions and camera used for the setup. It is recommended to optimize settings for a single DNA-PAINT measurement and apply the same parameters to a SUM-PAINT experiment. Generally, a 2D measurement requires a smaller box size than a 3D measurement due to the elliptical point-spread function.
  c. Press the “Localize (Identify and Fit)” button and Picasso will generate a hdf5 file containing all fitted localizations with their respective properties as well as an adjacent yaml file containing μManager and Picasso processing information.
  d. Proceed until all protein targets are localized.
31. Image drift-correction with ‘Picasso: Render’.
  a. Start ‘Picasso: Render’ and open the hdf5-file of one protein.
  b. Go to the button “Postprocess” and click on the button “Undrift by RCC”. Select 500 for segmentation and press “OK” to initiate the drift correction via redundant cross-correlations.
    ***Note:*** The segmentation parameter depends heavily on the number of frames of the entire movie stack of one protein target. For further details refer to Schnitzbauer et al.^2^
  c. Start selecting gold nanoparticles for drift-correction by selecting “Pick” under “Tools” and setting the pick size to 3 pixels in the “Tool Settings” dialog.
    ***Note:*** A pick size of 3 equals to 390 nm (depending on the pixel size of the camera), which should cover a gold nanoparticle after RCC drift correction. If this is not the case, a bigger pick size needs to be selected.
  d. Under “View”, “Display settings” change the Blur to one-pixel blur, which better highlights the gold nanoparticles.
  e. Select at least three gold nanoparticles and press the “Pick similar” button in the “Tools dialog”, which will automatically detect similar structures.
  f. Undrift the image via gold nanoparticles by pressing “Undrift by picked” in the “Postprocessing” dialog.
  g. Proceed until all protein targets are drift corrected.
32. Image alignment.
  a. Start ‘Picasso: Render’ and open the hdf5 file of all proteins.
  b. Go to the “Postprocessing” dialog and press the “Align channels” button.
  c. If necessary, repeat the step with picked gold nanoparticles similar to Step 31.

***Note:*** Alignment by RCC in Picasso can be unsuccessful when aligning a large number of datasets. In case of miss-alignment, it is recommended to align in smaller subsets (e.g. 6 proteins at a time).

### Synapse heterogeneity analysis

**Timing: Typically, this is an iterative process with several refinement steps taking several days to several weeks**

**Figure 3:**
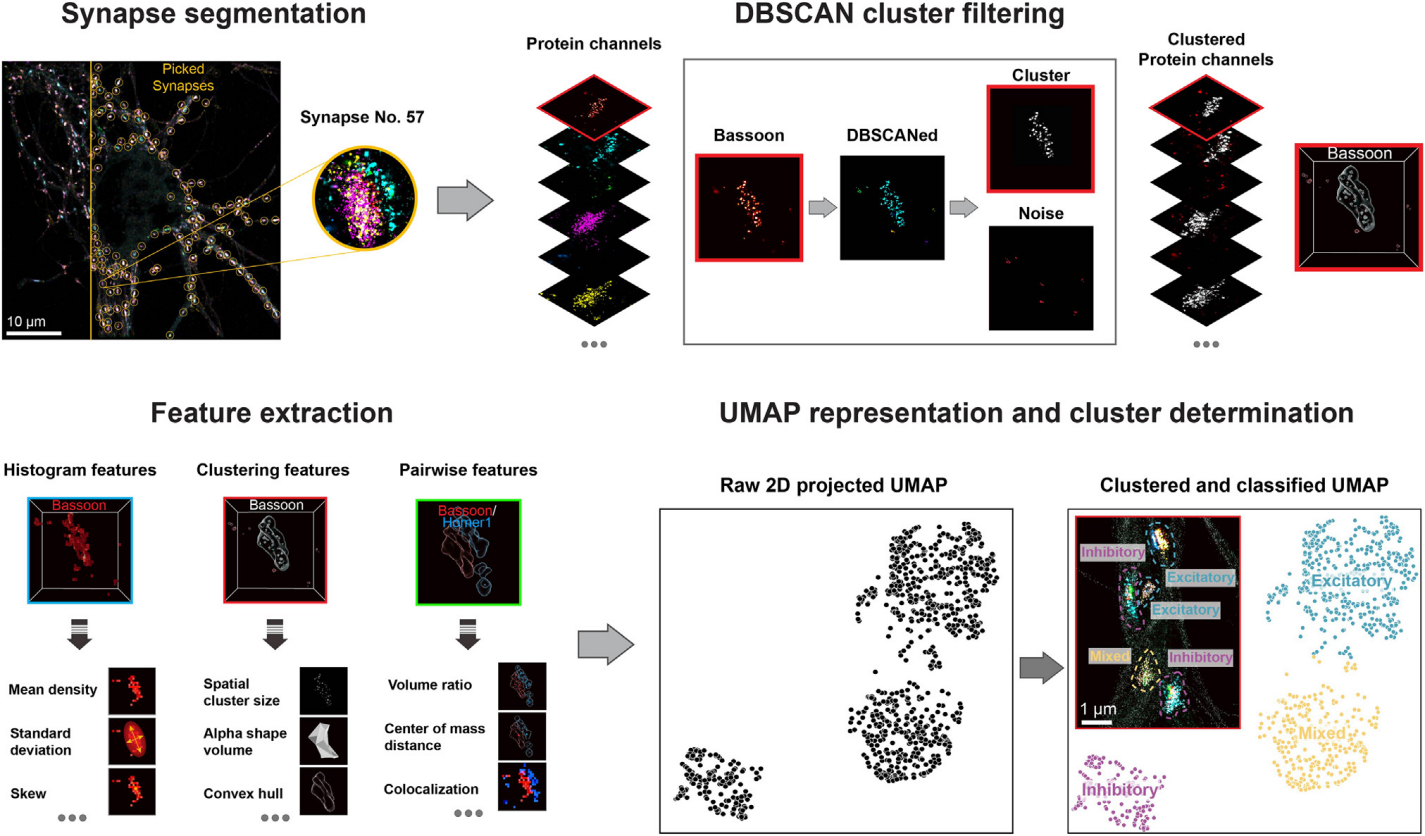
Workflow for synapse image analysis Steps describing synapse segmentation, feature extraction and unsupervised data exploration. Synapses are segmented by the overlay of synaptic marker channels and picked with Picasso.^2^ For clustering feature extraction, the synapse protein channels are DBSCANed to remove noise. Histogram, clustering and pairwise comparison features are extracted by custom analysis scripts from Unterauer et al.^1^ All features are projected into a 2-dimensional representation (UMAP) and after clustering in the UMAP, unsupervised data exploration can be performed.

In this section, the steps for synapse analysis are described in detail (Figure 3). In Unterauer et al.,^1^ the synapse selection (determining synaptic regions by the colocalization of synaptic markers) was performed in Picasso, the DBSCAN clustering and feature extraction was fully automated using the analysis script in the zenodo repository. Here, all steps for assembling the analysis script are described in general and can be implemented in a custom script. The resulting parameter vectors for each synapse can then be compiled with a dimensionality reduction algorithm (in Unterauer et al.^1^ UMAP^11^ was used). While the dimensionality reduction tools are widely used in omics-based analysis, it is important to point out that any feature should first be investigated and verified independently before compiling in a dimensionality reduction pipeline. In Unterauer et al.^1^ a broad selection of morphometric features is investigated but these features only serve as a suggestion. Generally, any feature of interest that can be computed out of the neuronal atlas can be added to the analysis workflow.

33. Synapse segmentation.
  a. Open ‘Picasso: Render’ and load synaptic scaffolds and associated vesicle pool protein channels for synapse selection.
    ***Note:*** In Unterauer et al.^1^ Bassoon, Homer1, Gephyrin, VGlut1, VGAT and Synapsin were overlaid in this preselection approach.
  b. Open the “Pick” tool as described in Step 31 and select a Pick size of 10 corresponding to 1.3 μm (depending on the pixel size of the camera).
  c. Select synapses interactively based on the colocalization of protein signal in scaffold and vesicle pool protein channel with the “Pick” tool.
  d. Go to “File”, “Save Pick Regions” to export a yaml file with the positions of the synapses.
  e. Load all proteins channels that are considered in the synaptic analysis.
    ***Note:*** In Unterauer et al.^1^ 13 proteins were considered at this step
  f. Load the pick region coordinates by going to “File”, “Load pick regions”.
  g. Export the localizations in the picked areas by pressing the “Save picked localizations” button and selecting “apply to all channels sequentially”.
  h. DBSCAN the exported synapse localizations to separate the synaptic clusters from the surrounding background signal.
    ***Note:*** There are various methods for implementing DBSCAN or other clustering techniques to distinguish synaptic cluster localizations from the surrounding background. A key aspect of this implementation is the precise determination of the separation cutoff, which is critical for selecting the structures to be further analyzed. While experts can manually assess each synapse and protein channel, Unterauer et al.^1^ optimized the DBSCAN parameters—such as clustering radius and localization cutoff—by first evaluating synaptic marker proteins and then comparing the characteristics of synaptic regions to randomly assigned regions within the cell.
    i. Load a synaptic marker channel (e.g., Synapsin) into ‘Picasso: Render’.
    ii. Go to “Postprocessing”, “Clustering”, “DBSCAN” and run the clustering with the parameters set to 80 nm radius and a density of 5 localizations.
      ***Note:*** Radius and density for DBSCAN depend partly on the image acquisition and the target structure. A longer image acquisition will result in more localizations and hence a higher density needs to be chosen and vice versa. The clustering radius is dependent on the target proteins and their distribution. For synaptic scaffold proteins, a slightly lower radius is recommended (e.g. 65 nm). Generally, it is recommended to troubleshoot many parameter combinations in the “Test clusterer” window until optimal parameters (separation of the synaptic cluster from outer noise) are found.
    iii. Filter the resulting DBSCAN cluster by cluster size.
      ***Note:*** In Unterauer et al.,^1^ the cluster size cutoff was determined by evaluating the cluster size of randomly picked, non-synaptic regions in the cell. Depending on the quality and specificity of the antibody, DBSCAN can be applied to these random regions using the same parameters as those used for synaptic regions. The largest clusters detected in these random regions can then serve as a threshold to distinguish true synaptic clusters from noise localization clusters.
    iv. Repeat until all protein targets are DBSCANed and filtered.
34. Synaptic feature extraction.
  a. Histogram feature calculation.
    i. Load the raw synaptic localization data (before DBSCAN cluster filtering) into the analysis script.
    ii. Calculate the center position of each synapse by arithmetic mean, with summing over the respective coordinate (x, y, z) and dividing by the number of points (e.g., (1/n) sum(x_i) for i = 1 to n).
    iii. Calculate histogram representation of each synapse with a diameter of 10 pixel (equal to the pick size) and 20 bins using the center as origin.
    iv. Calculate “histogram features”: Mean, standard deviation, skewness, kurtosis, entropy and intensity min and max.
    v. Calculate “histogram distances”: Wasserstein distance, Jensen-Shannon distance, Cosine distance and the pairwise histogram maximum distance (calculated by the difference of the argmax values of the histogram bins).
      ***Note:*** In Unterauer et al.,^1^ a custom script is used to extract features using the python packages Numpy,^12^ scikit-learn^13^ and SciPy.^14^ The respective analysis script is part of the full analysis script of Unterauer et al.^1^ and attached in the custom analysis script as histogram_features.py. It is possible to calculate these features with any other coding language or even manually inserting them.
      ***Note:*** In Unterauer et al.,^1^ only pairwise proteins were considered in the “histogram distance” features. For the design of a new custom analysis script, it is possible to extend the analysis to three-way or even all protein interconnecting features at this step.
  b. Clustering feature calculation.
    i. Load the DBSCAN clustered localizations into the analysis script.
    ii. Calculate the center of mass of all synapse localizations by the mean of all protein x, y and z coordinates.
    iii. Calculate the center of mass of the DBSCAN cluster size filtered localizations by the mean of x, y and z coordinates for each individual protein.
    iv. Calculate clustering features: number of localizations (before cluster size filtering in Step 33), largest cluster size (after cluster size filtering in Step 33), standard deviation (before cluster size filtering), convex hull (a volume estimate) and pairwise volume ratio (as volume of protein 1 divided by volume of protein 2).
    v. Calculate cluster distance features: pairwise center of mass distance between protein species and center of mass distance from each protein to the center of mass of all synaptic localizations.
    vi. Calculate alphashape features: Alphashape volume, localization density (as number of localizations over volume and surface area (area function of the alphashape package).
      ***Note:*** At this step in Unterauer et al.,^1^ two custom scripts are used to extract clustering and alphashape features using the python packages numpy, scikit-learn, scipy and alphashape. The respective analysis script is part of the whole analysis script of Unterauer et al.^1^ and attached in the custom analysis script as clustering_features.py and alphashape_features.py. It is possible to calculate these features with any other coding language or even manually inserting them.
      ***Note:*** In Unterauer et al.,^1^ only pairwise proteins were considered in the “cluster” and “cluster distance” features. For the design of a new custom analysis script, it is possible to extend the analysis to three-way or even all protein interconnecting features at this step.
  c. Compile all features in a csv (or storage file of choice).
    ***Note:*** For further processing using python, it is important to not assign NaN values for empty features (e.g. a Homer1 volume for an inhibitory synapse, which should be equal to 0). Make sure to assign 0 or other defined values to those otherwise NaN values.
35. UMAP dimensionality reduction and clustering.
  a. Preprocess the compiled feature list.
    i. Remove Zero-variance features by removing all features that have a standard deviation of zero across all synapses.
    ii. Apply Z-score normalization by subtracting the mean and dividing by the standard deviation for each feature value.
      ***Note:*** If working with multiple datasets, dataset specific biases can be introduced. In Unterauer et al.^1^ Z-score normalization was done dataset independently and the datasets were combined afterward for UMAP plotting.
  b. Create the UMAP model.
    i. Use the UMAP function to create a model. Set parameters to n_components = 2 and choose random_state as a seed for reproducibility.
    ii. Plot the UMAP as a scatterplot, the UMAP function returns a x and y component.
    iii. Optionally adjust the min_dist between points (default is 0.1) or choose a different random_state for better visualization.
      ***Note:*** When working with a large feature space it is crucial to step-wise determine the feature reliability and their meaning. In Unterauer et al.^1^ after plotting all 1590 features, the features were plotted grouped by their feature classes (e.g. clustering features, histogram features) to see individual contributions. Additionally, different combinations of protein features were plotted to see how these contribute to the separation (e.g. the three scaffold proteins, Bassoon, Homer1 and Gephyrin).
  c. Separate clusters in the UMAP.
    i. Perform clustering (e.g., K-means clustering) for a range of different cluster numbers.
    ii. Use the scikit-learn.metrics function silhouette_score to determine the similarity of data points to its own cluster points in comparison to other clusters.
    iii. Choose the number of clusters with the highest silhouette score for the cluster separation in the UMAP.
      ***Note:*** There are several metrics to determine the optimal cluster number in a UMAP. In Unterauer et al.^1^ the silhouette score and the elbow methods were used and yielded the same results. It is highly recommended to use several metrics and to test different clustering algorithms for optimal separation.
  d. Use the cluster information for unsupervised data exploration.
    ***Note:*** In unsupervised data exploration, dimensionality reduction techniques are employed to uncover patterns and relationships within the data, helping to identify similarities and differences. In the study by Unterauer et al.,^1^ UMAP revealed three distinct clusters, which was unexpected given the anticipation of only two primary synapse classes (excitatory and inhibitory). To investigate the origin of this unexpected separation, various features were visualized either through ensemble data representations or by applying a color gradient to the UMAP, highlighting specific features. By correlating the extent to which these features or groups of features influence the separation in UMAP, it is possible to identify the key drivers of this clustering. In the case of Unterauer et al.,^1^ the critical factors were the synaptic type marker proteins: Homer1, Gephyrin, VGlut1, and VGAT.

## Expected outcomes

Following this protocol, it is possible to generate a single-molecule resolved neuronal atlas for the number of proteins desired (in Unterauer et al.^1^ the distribution of 30 proteins is mapped). Each protein channel should have a localization precision of better than 10 nm and there should be no crosstalk between protein targets. The labeling efficiency, and consequently the detected signal for each protein channel, should be comparable to that of individual DNA-PAINT measurements. All targets in the atlas should be aligned with nanometer accuracy indicated by the overlay of gold nanoparticles among all protein channels. By applying the proposed analysis pipeline, single synapses are segmented out of the image and morphometric features can be extracted. The desired feature list (in Unterauer et al.^1^ there are in total 1590 features) can be analyzed individually (or in feature groups e.g., clustering features or cluster size for proteins a, b, c) or compiled in a dimensionality reduction setting as for example UMAP. The combination of the super-resolved atlas and the ensemble feature representation offers the possibility to explore heterogeneity among the synapse classes, while the neuronal atlas also allows to explore the cellular environment in general.

## Quantification and statistical analysis

Localization precision analysis should be done after removing gold nanoparticles and is performed in Unterauer et al.^1^ by using nearest neighbor-based analysis (NeNA^15^). Quantification of DNA-PAINT localizations are specific to the binder e.g., antibody in use and can usually not be compared towards other protein species (e.g., the localization numbers for Bassoon will differ from the ones detected with Homer1). A quantification of localization signal for identification of underlying protein copy numbers can be done following a qPAINT^16^ protocol. Single-molecule counting can only be done when the synapses for comparison are within the focal plane, which is typically a small fraction due to the 3D architecture of synapses bordering the small focal volume of the imaging setup. Note that the resolution for imaging synapses in HILO is typically not homogeneous among the whole z range. For quantification and comparison among protein species it is necessary to use a more unbiased factor, which in Unterauer et al.^1^ was set to be the protein volume. Imaging metrics are the localization precision and the specificity of the target. In Unterauer et al.^1^ the localization precision for all protein species was approx. 6 nm and signal extinction tests were employed to ensure secondary-label specificity (with 99.5% signal extinction when switching from one protein target to the next, calculated based on the localization counts) as well as a qualitative comparison between standard DNA-PAINT and SUM-PAINT individual protein target results. It is highly recommended to exclude data that have a localization precision worse than 8 nm (except for Lifeact imaging), does not show sufficient secondary-label specificity (worse than 99.5% extinction) or show signal differing from the individual DNA-PAINT imaging experiments.

## Limitations

The success of DNA-PAINT, and its extension SUM-PAINT, is highly dependent on the quality of the affinity reagents used. In Unterauer et al., we provide a comprehensive selection list not only for the 30 targets examined in the study but also for a wide range of DNA-PAINT-compatible binders. Although these reagents worked well across multiple experiments, it is essential to test newly acquired affinity reagents with DNA-PAINT, as different antibody batches may vary in performance. In Unterauer et al., secondary nanobodies were employed for immunostaining. While these secondary nanobodies offer significant flexibility when evaluating large numbers of binders, the preincubation strategy requires careful optimization of the concentration ratio between primary antibodies and secondary nanobodies. This optimization is crucial in Step 4 of the protocol to prevent crosstalk between channels.

The proposed imaging setup allows for the acquisition of approximately a 1 μm z-slice of the neuronal sample, with the prerequisite of the imaging plane being close to the coverslip. This is because HILO, a variation of selective plane illumination, can only capture images up to a maximum of approx. 5 μm from the coverslip without significant resolution degradation. The achievable resolution is also dependent on imaging time, as the highest precision DNA-PAINT requires sparse sampling to minimize imager crosstalk. In the study by Unterauer et al.,^1^ a balance was struck between rapid acquisition and optimal resolution. If higher resolution is required, longer imaging times should be considered.

## Troubleshooting

### Problem 1

DNA-PAINT images for individually acquired protein targets do not have sufficient resolution and/or too much background noise (see 30-plex SUM-PAINT image acquisition, step 10, 16, 17)

### Potential solution

**Figure 4:**
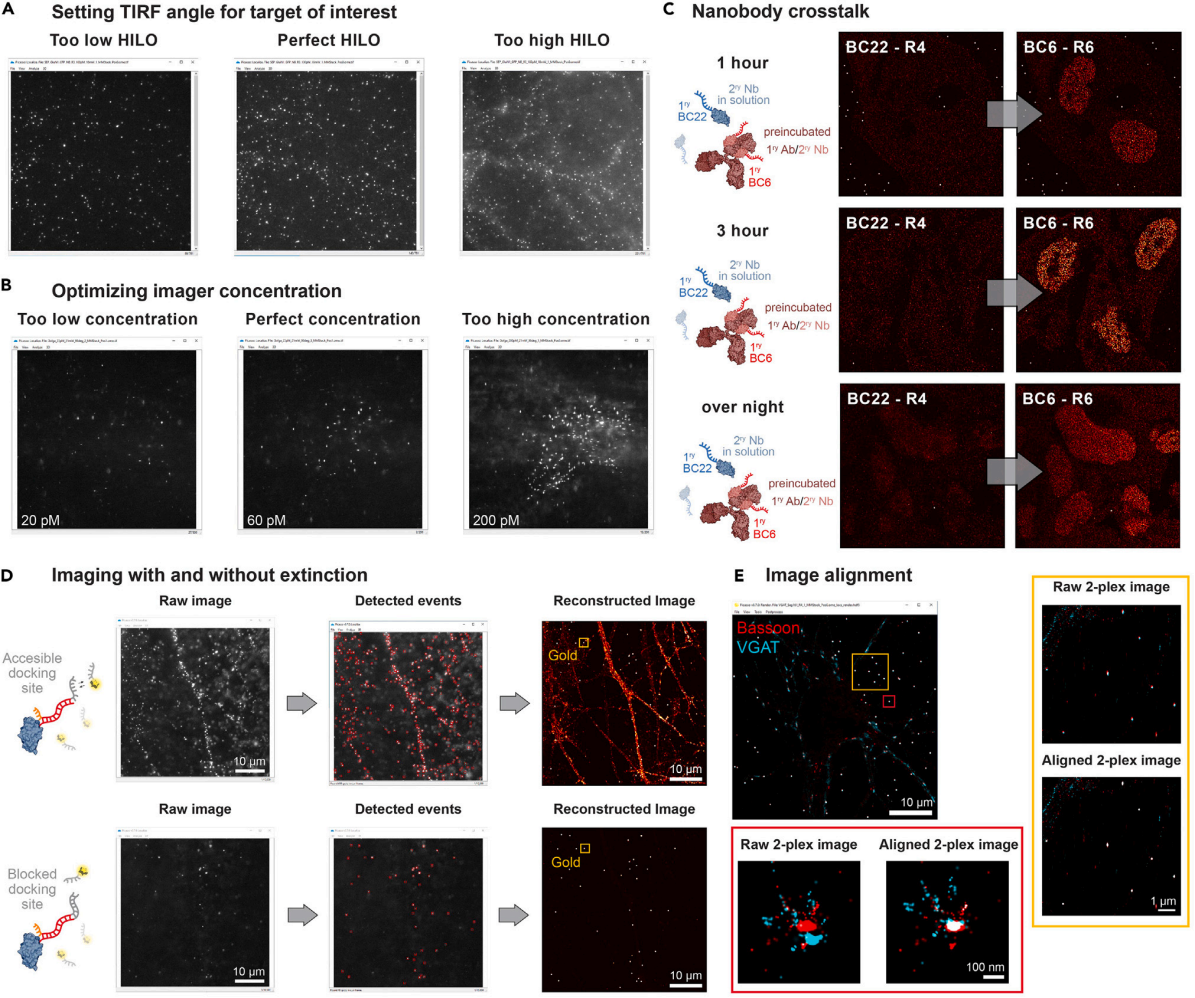
Problems and their possible solutions (A) Three TIRF angles for image acquisition of synaptic targets are shown in ‘Picasso: Localize’^2^ with the left image displaying a too low HILO sheet for image acquisition indicated uneven illumination and a clear loss of localization on the right side of the image. The middle image shows the perfect HILO illumination showing evenly distributed localizations throughout the field of view and continuous sampling. The right image shows a too high HILO with substantially higher noise and highly reduced sampling. HILO adjustment in detail in Video S1. (B) Three imager concentrations (20 pM, 60 pM, 200 pM, Videos S2, S3, and S4) for Golga5 acquisition are shown. 20 pM as imager concentration is too low indicated by very few blinking events. 60 pM is the optimal imager concentration for imaging Golga5 as the sample is regularly sampled with rarely overlapping point spread functions (PFS). 200 pM are substantially too high as several PSFs overlap. This imaging is useful for determining the perfect plane for target acquisition but should be replaced with a lower concentration for higher resolution. (C) Nanobody crosstalk qualitative comparison after 1 and 3 h and overnight (approximately 16 h) incubation of a sample with preincubated BC6 2ry nanobody to a 1ry antibody and BC22 nanobody in solution. Imaging of the same region indicates that there is no visible crosstalk after 3 h but a minor part appears with overnight (approximately 16 h) incubation. (D) Comparison of emission detection before and after extinction. The top row shows SUM-PAINT imaging before extinction. The raw image as well as the PSF detection show a highly sampled structure, which is reconstructed afterward. In the bottom row the same region after extinction by blocking shows only gold detection and the reconstructed image shows no trace of an underlying structure. (E) Image alignment workflow in ‘Picasso: Render’. The raw overlay of two regions shows an offset of a few 100 nm. Image alignment by Picasso results in nanometer precise overlay.

Optimal image acquisition for DNA-PAINT is dependent on multiple parameters. First, the microscope setup needs to be a current state-of-the-art single-molecule localization-based super-resolution setup. Here it is recommended to use a state-of-the-art sCMOS camera (e.g., Andor Zyla 4.2 or Hamamatsu Fusion BT). Secondly, DNA-PAINT image acquisition is usually done relatively close to the coverslip surface. The achievable resolution is dependent on the plane illumination mode. Typically, in TIRF mode, 8 nm resolution in cells is possible, while when imaging in HILO, target resolution scales inversely with the distance from the coverslip. This is due to the fact that the illumination volume is substantially larger in HILO and background imagers will contribute more to the signal to background ratio. Setting the HILO illumination for optimal illumination of the focal plane is crucial for best image quality. Figure 4 A shows three different settings for the TIRF angle corresponding to a too low HILO, HILO in focus and a too high HILO (see also Video S1). Lastly, DNA-PAINT image quality also depends on the optimal imager concentration for the specific target of interest. A too low imager concentration will result in insufficient sampling, requiring long imaging times, eventually leading to sample degradation. A too high imager concentration results in localization detection crosstalk and resolution will decrease substantially. Figure 4B shows Golga5 (Golgi apparatus) acquisition with too low, optimal and too high imager concentration (see also Videos S2, S3, and S4). For the specific problem of imager pre-bleaching and high signal to background, fluorogenic DNA-PAINT can be a possible solution, since imagers are only fluorescent when binding to the target of interest.

Video S1. HILO adjustment for imaging of synaptic targets, related to Problem 1

Video S2. Golga5 imaging with 20 pM imager concentration, related to Problem 1

Video S3. Golga5 imaging with 60 pM imager concentration, related to Problem 1

Video S4. Golga5 imaging with 200 pM imager concentration, related to Problem 1

### Problem 2

DNA-PAINT images show no specific signal, even though confocal imaging with the respective primary antibody and secondary nanobody is working (see 30-plex SUM-PAINT image acquisition, step 17).

### Potential solution

Being coupled to a DNA strand, potential interaction most likely due to the negative charge of the DNA can influence the binding properties of the antibody to the protein target. If Problem 2 occurs, it is recommended to first test the affinity reagents in a single protein measurement, to evaluate if the target can be imaged without preincubation of primary antibody to secondary nanobody. If the target shows specific signal, SUM-PAINT experiments need to be designed in a way that the problematic target is imaged in a separate initial imaging round, followed by unconjugated nanobody blocking, before the outlined protocol can be followed. Should this not lead to success, a different secondary affinity reagent can be tested. E.g. NanoTag Biotechnologies GmbH offers more variants of mouse secondary nanobodies, or it is possible to use a secondary antibody for the specific target of interest. Should none of the proposed solutions work, the target either needs to be discarded from the SUM-PAINT codebook, or a site-specific tag needs to be introduced (e.g., GFP or ALFA-tag) to enable imaging of the target using tag-specific DNA-PAINT nanobodies.

### Problem 3

SUM-PAINT images show crosstalk between protein channels (see sample preparation for DNA-PAINT imaging, step 4 and 30-plex SUM-PAINT image acquisition, step 18).

### Potential solution

When protein targets show crosstalk in overlaid image reconstruction, it is likely due to an imbalance in primary antibody to secondary nanobody concentration. It is recommended to optimize the concentration ratio by a two-target preincubation measurement in which the nanobody to antibody ratio and concentration is varied until both targets show sufficient staining and no cross-talk. For example, if Neurofilament (mouse primary AB 1:300, secondary nanobody kLC 1:300) shows crosstalk signal with the VAMP2 (species mouse 1:300, secondary nanobody kLC 1:300) channel, the secondary nanobody concentration for Neurofilament can be adjusted to 1:400. In addition, the unconjugated nanobody concentration and incubation time for blocking can be adjusted. Lastly, the incubation time can be shortened as the probability for crosstalk increases with prolonged incubation time.

### Problem 4

Signal extinction by blocking and/or toehold-mediated strand-displacement does not remove the protein signal efficiently (see 30-plex SUM-PAINT image acquisition, step 18 and step 22).

### Potential solution

Signal extinction is dependent on the hybridization efficiency of the respective strands, which can be compromised in regions with low accessibility. If problem 4 occurs, it is recommended to test reagents in an *in vitro* measurement (e.g., DNA origami) or a single-target protein acquisition. Strand concentration and incubation time can be prolonged for a higher extinction efficiency. If problem 4 persists, the respective strands should be reordered and fresh aliquots should be used for testing. If problem 4 only occurs for one specific target, it is recommended to image this target in the last barcoding round, as blocking is not necessary after that round. Lastly, docking and blocking strands can be elongated by 1 or 2 base pairs to make the blocking process more stable and increase blocking efficiency.

### Problem 5

Image alignment with Picasso shows substantial offset (see super-resolved image reconstruction and neuronal atlas alignment, step 32).

### Potential solution

Image alignment depends mainly on the gold nanoparticles imaged in the field of view. If there are too few gold nanoparticles (usually below 3), it is highly recommended to increase the incubation ratio of gold nanoparticles to PBS in Step 3. If alignment by RCC is not working, alignment can be done by manual selection of gold nanoparticles as alignment structures (by selecting them with the “Pick” tool). Lastly, image alignment can be problematic when aligning large numbers of different protein channels. It is recommended to use the first protein target as reference and align the remaining proteins in set of approximately 6 targets.

### Problem 6

DBSCAN clustering is not segmenting the synaptic protein target (see synapse heterogeneity analysis, step 33).

### Potential solution

The DBSCAN input parameters depend on several factors: the resolution of the image, the specificity of the antibody (as specific signal to noise), the density of the protein structure and sufficient sampling of the molecules. If the resolution is worse than 20 nm, it is recommended to refer to problem 1 and repeat the measurement or discard the protein target for the analysis. If the image shows substantial non-specific background signal, synapses can be individually selected with the pick tool “Polygon” to avoid noise input into the DBSCAN clustering pipeline. Another possibility is to put stricter cutoff values in the cluster filtering steps. Using the noise regions as reference might not be strict enough and the localization cutoff can be increased. Lastly, clustering parameters can be varied for individual synapses until the desired clustering result is achieved. Note that this step should be done with respect to marker proteins (e.g., Synaptotagmin for the vesicle pool, or Bassoon for presynaptic scaffold) to avoid any biased selection by the experimenter.

## Resource availability

### Lead contact

Further information and requests for resources and reagents should be directed to and will be fulfilled by the lead contact, Ralf Jungmann (jungmann@biochem.mpg.de).

### Technical contact

Technical questions on executing this protocol should be directed to and will be answered by the technical contact, Ralf Jungmann (jungmann@biochem.mpg.de).

### Materials availability

This study did not generate new reagents.

### Data and code availability

No original code has been generated in this study. All data and code were taken from Unterauer et al.^1^ and are available at Zenodo: https://doi.org/10.5281/zenodo.10212680.

## Acknowledgments

This research was funded in part by the European Research Council through an ERC Consolidator Grant (ReceptorPAINT, grant agreement number 101003275), the BMBF (Project IMAGINE, FKZ: 13N15990), the Max Planck Foundation, and the Max Planck Society.

E.M.U. and E.-M.S. acknowledge support by the IMPRS-ML graduate school. F.O. acknowledges support by Deutsche Forschungsgemeinschaft (DFG) through the SFB1286 (project Z04). E.F.F. is funded by a CZI collaborative pair grant. E.F.F. also acknowledges the support of the Collaborative Research Center 1286 on Quantitative Synaptologie (CRC/SFB1286), Göttingen, Germany. S.S.B. has received funding from F. Hoffmann-La Roche LTD (no grant number is applicable) and is supported by the Helmholtz Association under the joint research school “Munich School for Data Science - MUDS.” C.M. has received funding from the European Research Council (ERC) under the European Union’s Horizon 2020 research and innovation program (grant agreement no. 866411). Figures were created with the help of BioRender (https://biorender.com).

## Author contributions

E.M.U., E.-M.S., and K.J. shared conceptualization, discussion, writing, and editing and contributed equally to the manuscript. S.S.B. outlined the data analysis workflow. R.J. and E.F.F. supervised the project and edited the final version of the manuscript. All authors reviewed and approved the final manuscript.

## Declaration of interests

E.M.U. and R.J. have filed a patent on the method described in this paper. F.O. is a shareholder of NanoTag Biotechnologies GmbH.
