# Supplementary material for: Protocol for SUM-PAINT spatial proteomic imaging generating neuronal architecture maps in rat hippocampal neurons
Source: STAR Protoc. 2025 Mar 5;6(1):103637. doi: 10.1016/j.xpro.2025.103637 (PMC11928808; doi:10.1016/j.xpro.2025.103637)
Supplement: Document S1. Figure S1 [file mmc1.pdf]

Supplemental Figures

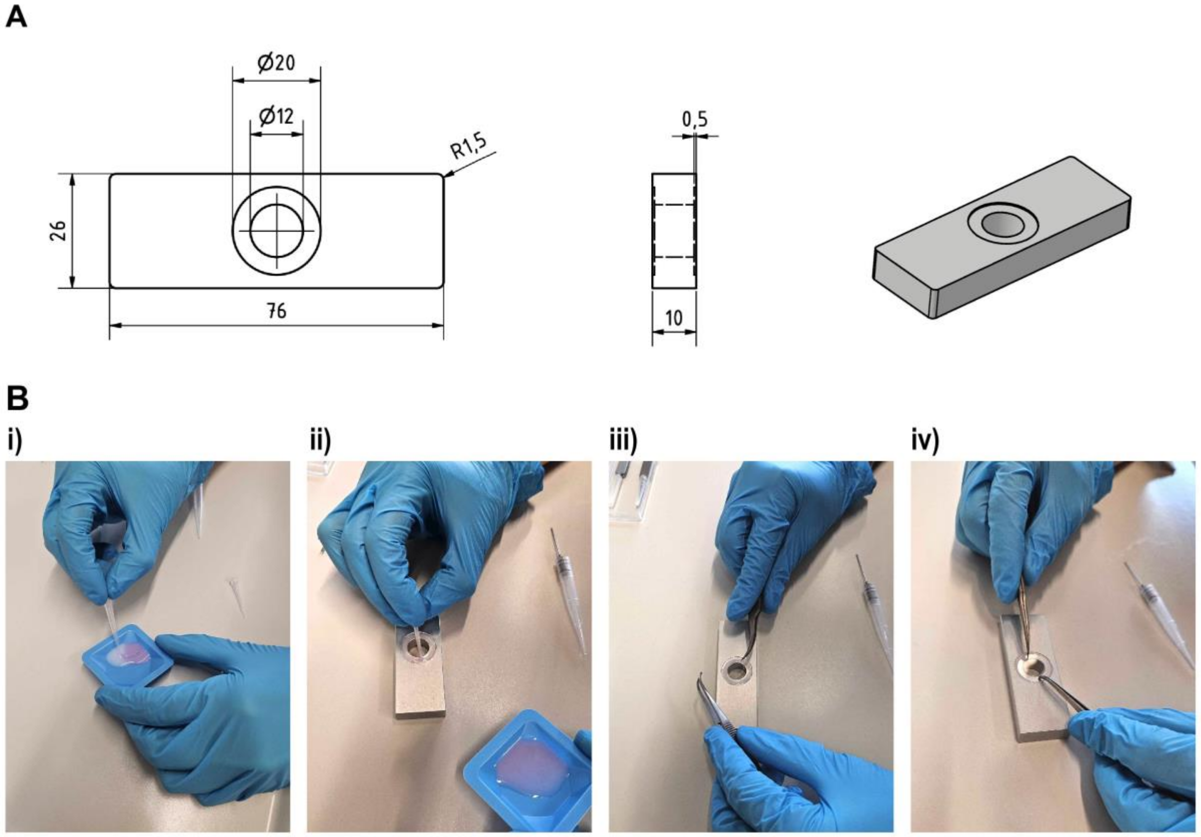

Figure S1: Sample Chamber Design and Preparation Workflow for Experimental Setup, related to Step 1
(A) Schematic illustration of the custom-made aluminum sample chamber used in the protocol. The chamber measures 76 mm in length, 26 mm in width, and 10 mm in height, with a central circular ring of 20 mm diameter and 0.5 mm depth, designed to hold an 18 mm glass coverslip. Additional measurements include a 12 mm inner opening to allow for buffer exchange. Note, that other sample holders that support coverslip mounting and buffer exchange may also be used.
(B) Step-by-step assembly process. (i) Preparation of Picodent Dental silicone glue by mixing equal parts for coverslip fixation. (ii) Even distribution of the silicone glue onto the central chamber ring using a 10 µL pipette tip. (iii) Placement of the coverslip onto the chamber ring using curved tweezers. (iv) Final pressing of the coverslip with two tweezers to ensure even glue distribution and complete sealing of the central chamber, allowing for liquid exchange. Gloves and sterile handling tools are used throughout the procedure to maintain experimental integrity.

Supplemental Videos

Methods Video S1: HILO adjustment for imaging of synaptic targets, related to Problem 1

Methods Video S2: Golga5 imaging with 20 pM imager concentration, related to Problem 1

Methods Video S3: Golga5 imaging with 60 pM imager concentration, related to Problem 1

Methods Video S4: Golga5 imaging with 200 pM imager concentration, related to Problem 1
